# Prediction of axillary lymph node metastasis in early breast cancer patients with ultrasonic videos based deep learning

**DOI:** 10.3389/fonc.2023.1219838

**Published:** 2023-09-01

**Authors:** Wei-Bin Li, Zhi-Cheng Du, Yue-Jie Liu, Jun-Xue Gao, Jia-Gang Wang, Qian Dai, Wen-He Huang

**Affiliations:** ^1^ Cancer Center and Department of Breast and Thyroid Surgery, Xiang’an Hospital, School of Medicine, Xiamen University, Xiamen, China; ^2^ Fujian Key Laboratory of Precision Diagnosis and Treatment in Breast Cancer, Xiamen, China; ^3^ Xiamen Key Laboratory of Endocrine-Related Cancer Precision Medicine, Xiamen, China; ^4^ Xiamen Research Center of Clinical Medicine in Breast and Thyroid Cancers, Xiang’an Hospital of Xiamen University, School of Medicine, Xiamen University, Xiamen, China; ^5^ Department of Ultrasonic Medicine Affiliated Hospital of Xizang Minzu University, Xianyang, China; ^6^ National Institute for Data Science in Health and Medicine, Xiamen University, Xiamen, China; ^7^ Department of Ultrasonic Medicine, Xiang’an Hospital, School of Medicine, Xiamen University, Xiamen, China; ^8^ Department of Ultrasonic Medicine of Shantou Central Hospital, Shantou, China; ^9^ School of Informatics, Xiamen University, Xiamen, China

**Keywords:** axillary lymph node metastasis, artificial intelligence, ultrasound video image, breast lesion, deep learning model

## Abstract

**Objective:**

To develop a deep learning (DL) model for predicting axillary lymph node (ALN) metastasis using dynamic ultrasound (US) videos in breast cancer patients.

**Methods:**

A total of 271 US videos from 271 early breast cancer patients collected from Xiang’an Hospital of Xiamen University andShantou Central Hospitabetween September 2019 and June 2021 were used as the training, validation, and internal testing set (testing set A). Additionally, an independent dataset of 49 US videos from 49 patients with breast cancer, collected from Shanghai 10th Hospital of Tongji University from July 2021 to May 2022, was used as an external testing set (testing set B). All ALN metastases were confirmed using pathological examination. Three different convolutional neural networks (CNNs) with R2 + 1D, TIN, and ResNet-3D architectures were used to build the models. The performance of the US video DL models was compared with that of US static image DL models and axillary US examination performed by ultra-sonographers. The performances of the DL models and ultra-sonographers were evaluated based on accuracy, sensitivity, specificity, and area under the receiver operating characteristic curve (AUC). Additionally, gradient class activation mapping (Grad-CAM) technology was also used to enhance the interpretability of the models.

**Results:**

Among the three US video DL models, TIN showed the best performance, achieving an AUC of 0.914 (95% CI: 0.843-0.985) in predicting ALN metastasis in testing set A. The model achieved an accuracy of 85.25% (52/61), with a sensitivity of 76.19% (16/21) and a specificity of 90.00% (36/40). The AUC of the US video DL model was superior to that of the US static image DL model (0.856, 95% CI: 0.753-0.959, P<0.05). The Grad-CAM technology confirmed the heatmap of the model, which highlighted important subregions of the keyframe for ultra-sonographers’ review.

**Conclusion:**

A feasible and improved DL model to predict ALN metastasis from breast cancer US video images was developed. The DL model in this study with reliable interpretability would provide an early diagnostic strategy for the appropriate management of axillary in the early breast cancer patients.

## Introduction

Breast cancer is the most commonly diagnosed cancer among female malignancies worldwide and has become the leading cause of cancer-related deaths (1). The involvement of axillary lymph nodes (ALNs) is a crucial prognostic factor for breast cancer patients, and accurate axillary staging is critical for evaluating the disease status. Once ALN metastasis occurs, the clinical stage and treatment regimen may change, which can significantly affect patient prognosis (2, 3). However, the diagnosis of metastatic ALNs from imaging can vary greatly depending on the imaging results of different-level physicians/radiologists. Currently, the standard procedure for diagnosing ALN status before surgery is pathological examination of the lymph node obtained through biopsy. Clearly, lymph node biopsy is an invasive procedure with relatively complicated operation and is time-consuming (4–6). Lymph nodes biopsy can probably cause complications such as soft tissue infection, bleeding, seroma. Occasionally, it will cause the subcutaneous effusion due to lymphatic fistula. In addition, the technique has a false-negative rate ranging from 7.8% to 27.3% (7–9).

General imaging studies a variety of non-invasive diagnostic tools, including ultrasound (US) (10), magnetic resonance imaging (MRI) (11), and mammography (12). Asian women, especially young women, have denser breast tissue (13), and US has been shown to be more suitable for the detection of breast lesions. Thus, US is widely used as the first choice for breast disease screening due to its convenience, non-invasiveness, real-time capabilities, absence of radiation, and flexibility in performing ultrasound-guided biopsies (14, 15). Usually, the US diagnosis of ALN is based on the size or shape of the lymph node and the status of the lymphatic gate, which can lead to significant variability due to the subjectivity of examination and the interpretation skills of ultra-sonographers (16). Previously, our group combined US findings and clinico-pathological factors and accurately predicted probability of ALN metastases. The model was further validated in a Dutch population with predictive probability less than 12%. However, this approach is still based on histopathological information including histological grade, hormone receptor and Her2 status obtained from either core needle biopsy or excision. Therefore, a new noninvasive method for rapid, accurate, and objective evaluation of ALN metastasis in breast cancer is urgently needed.

In recent years, artificial intelligence (AI) has been gradually applied in breast imaging to improve workflows, perform automatic image segmentation, enable intelligent diagnosis, and predict ALN metastasis accurately (17–19). To avoid the complicated feature extraction process and extract more abundant information from the image, the deep learning (DL) method has been widely used in medical image research in recent years (20, 21). DL transforms the original image data into a higher-level, more abstract expression through a hierarchical network and replaces the complex process of manual extraction and feature design by obtaining hierarchical feature information.

Current prediction models for ALN metastasis are based on static two-dimensional ultrasound images. Sun et al. manually delineated regions of interest (ROIs) on breast ultrasound images and constructed deep learning models based on intratumor, peritumor, and peritumor plus peritumor using the DenseNet network (22). The results showed that the three deep learning models had better prediction performance than their corresponding radiomics models. Additionally, peri-tumor microenvironment information can improve the accuracy of the ALN prediction model performance.

A recent study reported the effectiveness of DL models using three different networks to predict ALN metastasis in multicenter breast cancer patients. The results showed that the diagnostic ability of the DL model was also better than that of ultra-sonographers (23). In another study, Zheng et al. used deep learning radiomics of conventional ultrasound to extract depth features from static images and shear wave elastography images and established a deep learning image group model to predict ALN metastasis (24). The diagnostic performance in predicting ALN status between disease-free axilla and any axillary metastasis with an AUC of 0.902 (95% CI: 0.843-0.961). Therefore, deep learning has broad application prospects in predicting the status of ALN metastasis in breast cancer.

Previous studies were limited to analyzing ultrasound static (single frame) images instead of sequential image acquisition of video, which can cause the loss of many subtle or important US lesion features in the keyframe, and even some subtle lesions may be omitted (25). Additionally, hyperplastic glandular tissues or ribs may be mistaken for masses, which can mislead the deep learning model and result in misjudgment. Therefore, the aim of this study is to construct a non-invasive, rapid, accurate, and objective prediction model of ALN metastasis in breast cancer patients based on ultrasound video images. This model can guide the selection of clinical tumor staging and surgical methods, thereby avoiding unnecessary ALN biopsies in breast cancer patients. This study aims to develop a DL model using videos that demonstrate diagnostic performance comparable to that of experienced ultra-sonographers. Unlike previous studies that relied on static images, our model leverages the dynamic nature of video to capture more subtle features of the breast and surrounding tissues. By incorporating video data, we aim to improve the accuracy of ALN metastasis prediction and reduce the need for invasive procedures such as biopsy.

## Materials and methods

### Patients

**Figure 1:**
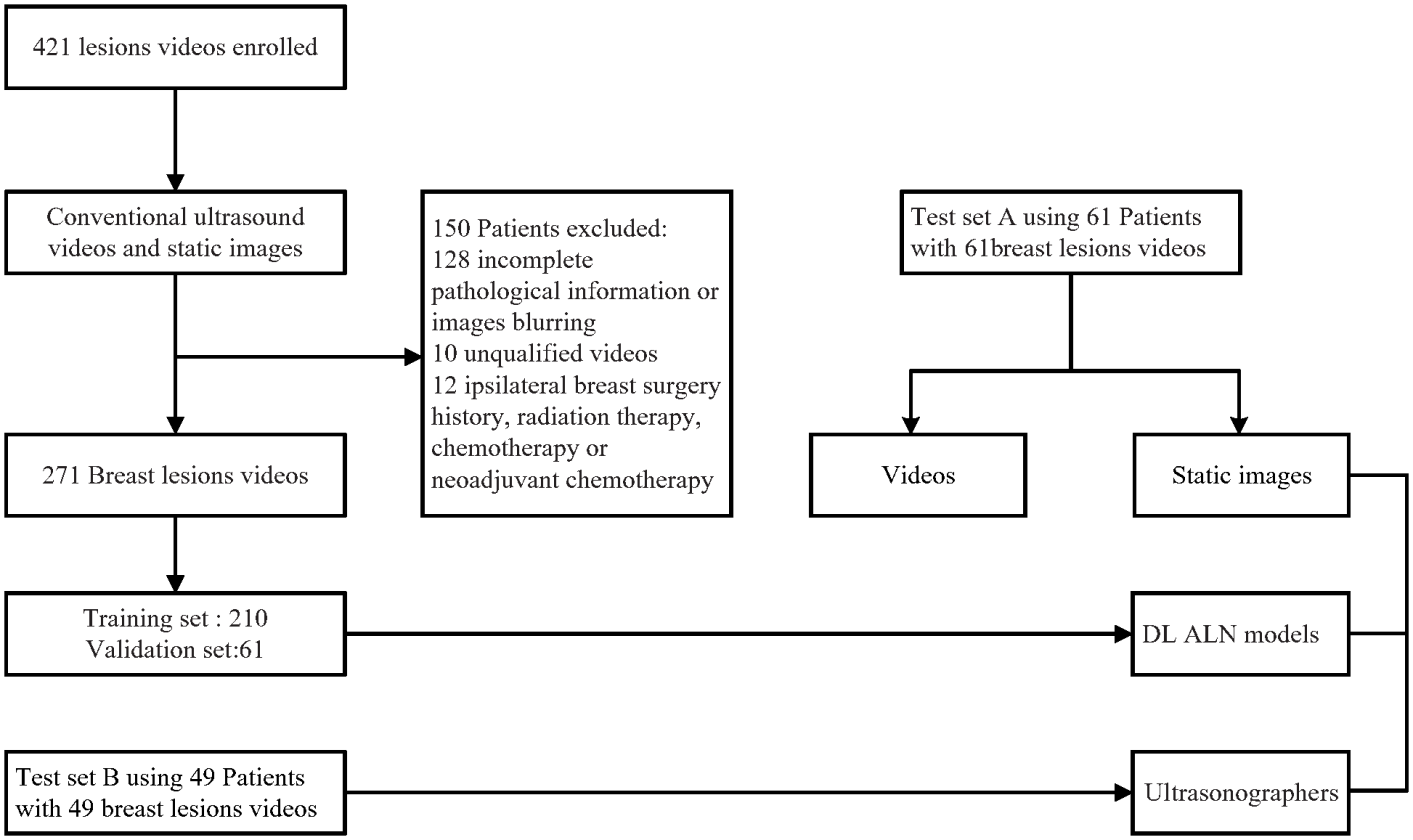
Patient recruitment workflow. In total, 271 US videos of 271 patients were included according to the inclusion criteria. The included patients were examined by conventional US and had complete clinical information for this study.

This retrospective multicenter diagnostic study was approved by the institutional review board of Xiang’an Hospital of Xiamen University and Shantou Central Hospital, informed consent was obtained from all participants (XAHLL2022060(2022) Research 041). Breast cancer US video data were acquired from 421 patients from Shantou Central Hospital, Guangdong, China, and Xiang’an Hospital of Xiamen University, Fujian, China, from September 2019 to June 2021. The inclusion criteria for the study were as follows: (a) patients with breast cancer confirmed by pathology; (b) clinical stage of T1-T2 and no distant metastasis; (c) complete US videos of the breast lesions were obtained; (d) patients without malignant tumors other than breast cancer; and (e) no history of axillary surgery. The exclusion criteria were (a) ALN involvement due to diseases other than breast cancer and (b) treatment for breast cancer with hormonal therapy, chemotherapy, or radiation therapy before surgery. The scanning and ultrasound diagnosis of the lesions was performed by ultra-sonographers experienced in breast ultrasound diagnosis by using the cross-plane screening method. Imaging data for the longitudinal and transverse views of each lesion were stored in the ultrasound equipment and then transferred to a hard disk for storage in a picture archiving and communications system (PACS). A flowchart describing the research process is shown in **Figure 1**.

Finally, 210 breast cancer US videos of 210 patients (121 without ALN metastasis and 89 with ALN metastasis) were used as the training and validation set, and the other 61 US videos of patients (34 without ALN metastasis and 27 with ALN metastasis) were used as testing set A. Another independent testing set B consisted of 49 breast cancer US videos of patients (38 without ALN metastasis and 11 with ALN metastasis) from Shanghai’s tenth Hospital of Tongji University, China, from July 2021 to April 2022.

### Data analysis

Ultrasound examinations were performed by five ultra-sonographers (two from Xiang’an Hospital of Xiamen University and three from Shantou Central Hospital) who had 8-20 years of experience in breast ultrasound. Ultrasound systems (GE Healthcare, USA; Siemens, Munich, Germany; Philips, Amsterdam, the Netherlands) were used to generate the ultrasound images. Image quality control was done for all videos, the L12-5 linear probe at a frequency of 12 MHz, the ultrasound images were adjusted by gain, focus, and zoom as needed. We used B-mode to examine the breast lesions and obtained transverse or longitudinal images. After performing 2D US, the videos and some single images with the fewest artifacts and the best quality was chosen and stored image for analysis and all breast US images extracted from systems were converted into DICOM format. Women with breast disorders were referred for breast ultrasound (BUS) and those with Breast Imaging Reporting And Data System, BI-RADS) ≥4 had ultrasound-guided biopsy and surgical resection.

### Deep learning model

All the clinical and pathologic data were obtained from the medical records. Pathologic results of the breast cancer included tumor clinical stage, tumor location, pathologic results of ALN status and tumor type. Clinical data included patients age, tumor size, BI-RADS category and pathologic results of ALN status with and without metastasis were recorded.

The original breast ultrasound data obtained from the clinic was relatively rough and there was a large amount of redundant information such as equipment parameters and other information around the imaging position. A series of pre-processing steps were adopted to normalize the data before training models. The steps were as follows: 1. Only the intermediate lesion position of the images was retained by cropping and excluding marginal irrelevant information and patient text data; 2. After adjusting the video to 256×256 pixels, various complex sampling situations are simulated through random horizontal flipping, elastic transformation, random cropping and other data enhancement methods to enhance the data variety and model generalization; 3. Scaled the video to 224×224 while the pixel values were normalized to 0 ~ 1. Through a series of data augmentation methods, the possibility that the input of the network was the same as the image seen by the network before each training was significantly reduced, and the diversity of data samples was further increased.

### Interpretability of DL model

**Figure 2:**
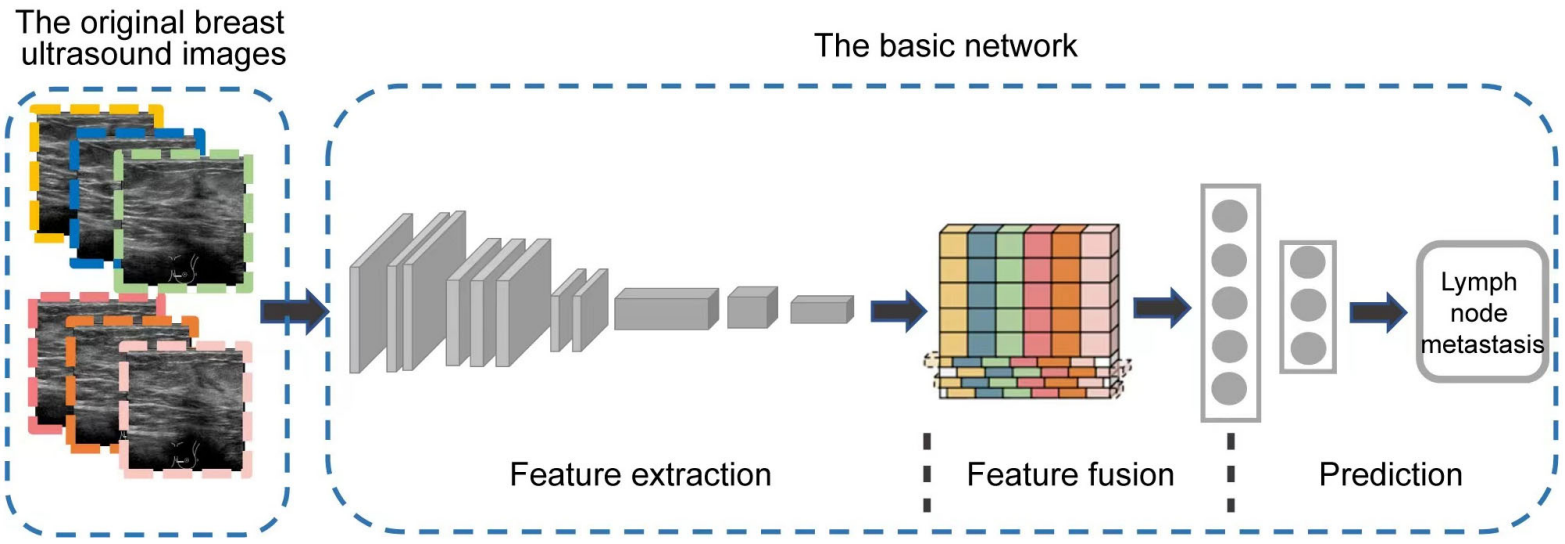
The overview of our DL model architecture. It mainly consists of three modules including feature extraction, feature fusion, and final classification prediction.


At present, CNNs are the most well-known type of DL in medical image analysis (26). Our DL model mainly consisted of three modules including feature extraction, feature fusion, and final classification prediction, using the classical CNN network ResNet-3D as the backbone (27), as shown in **Figure 2**. The basic network contained five stages. The first stage was the preprocessing stage for the input, including a simple convolutional layer and a max-pooling layer, and the subsequent four stages were composed of multiple residual blocks to realize the core residual structure in ResNet. In the Feature Fusion Module, unique interlaced offsets were provided for different frames to fuse spatiotemporal information (28). In the training, we utilized the widely adopted CrossEntropy Loss as the loss function in this study, given its extensive application in various classification tasks across the field. By employing the CrossEntropy Loss, which has become a standard choice in such scenarios, we aimed to effectively measure the disparity between predicted class probabilities and the true labels, thus facilitating accurate model optimization.

(1) $$H\left(p,q\right)=-\sum_{x}p\left(x\right)logq\left(x\right)$$

where *p* represents the true label and q represents the predict value.

The network and code of this study were written and implemented in Python using Pytorch. All deep learning algorithms were trained in the same training setting and trained on the Linux Server Ubuntu 18.04 LTS platform, GeForce RTX 3080 Ti GPU. During the training process, the network weights were randomly initialized and trained by the Adam optimizer, and the initial learning rate was set to 1.0×10^−4^. We also trained the model on static images for comparison, where the training process model was trained on static images, using the same testing set (testing set A: 61 patients) as the dynamic videos at inference time. 5-fold cross-validation was made to make the experiment results more reliable and accurate. The sample length T (Time channel) was set to 16 and the batch size was set to 4, for a total of 300 training epochs. We selected three CNN models of R2 + 1D (29), TIN (30) and ResNet-3D architectures for comparison and analysis. For static image training, we used three representative models ResNet, Inception V3, and VGG. The trained DL models outputted the predicted probability of the presence of lymph node metastasis according to US videos and chose the class of the highest probability as the prediction result. The results were measured based on the difference between the predicted output of the best model trained by five-fold cross-validation and the true labels of the testing set, and the corresponding accuracy, sensitivity, specificity, ROC curves, and PR curves were calculated. In addition, to better compare the performance of the models, we also compared the results of dynamic videos with static images. Temporal Interlace Network (TIN) presents a simple but effective module for video classification. TIN does not learn temporal features, but fuses spatio-temporal information by interleaving spatial features from past to future and future to past. A differentiable sub-module can calculate the offset of the interleaved features in the time dimension, and can rearrange the features according to the offset, so that each group of features is displaced by a different distance in the time dimension. Thus, 3D convolution is replaced by convenient and fast feature displacement operation to realize information exchange between adjacent frames. This makes the number of parameters and computation of the network much lower than that of ordinary 3D convolutional networks, making the network as a whole quite lightweight.

### Clinical application of DL model

To obtain ultra-sonographers’ predictive performance on the independent testing set, we performed an independent evaluation in testing set B. Three ultra-sonographers of different levels of experience in breast ultrasound were trained in how to perform predictive analysis based on some typical characteristics, such as the size of the lesions and the presence of lymphatic gate invasion (31), calcifications (32), architectural distortions (33), margin, cortical morphologic features, cortical medulla thickness, and blood flow (34, 35). The clinical real-time workflow consisted of three components. First, the ultra-sonographers were blinded to the pathological information of the axillary lymph node while they were scanning the axilla. Second, they analyzed some typical characteristics of breast cancer US images with the use of the American College of Radiology Breast Imaging Reporting and Data System (BI-RADS) (36). Third, the ultra-sonographers conducted a reexamination of the patient’s US video. They utilized the trained DL model to directly input the test image into the model, which provided an immediate output indicating the metastatic status of the ALNs. Finally, we compared the performance of ultra-sonographers with the DL model.

### Model interpretation

**Figure 3:**
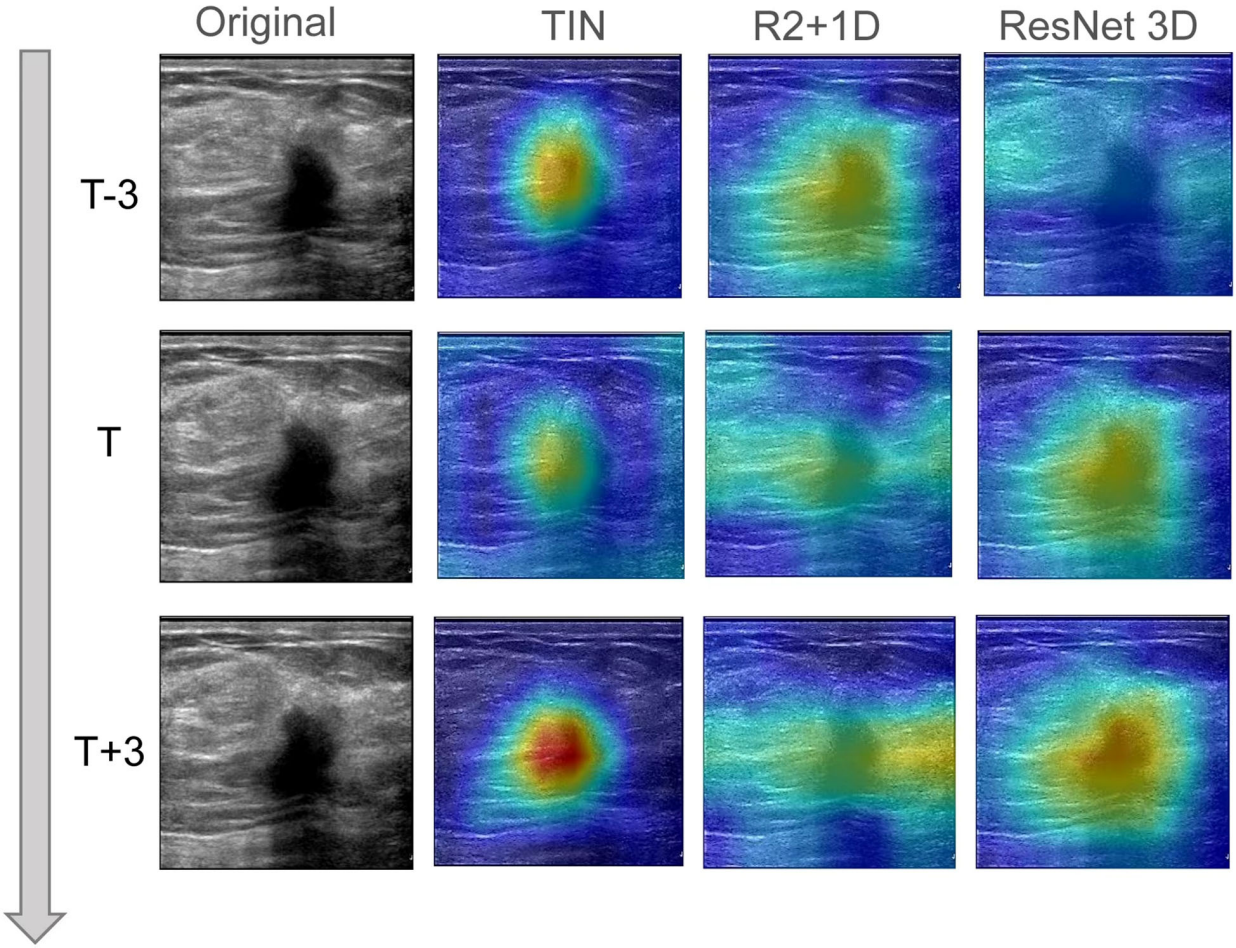
The visual class activation diagram of the prediction results of dynamic video models. The brighter the color, the better the visibility of the model. The best TIN model focuses more on the location of the nodules than the other two models, and the characteristics of the nodules are the most important basis for predicting whether the nodules will metastasize.

In this study, we utilized Grad-CAM technology to improve the interpretability of our models, which was crucial for clinical applications. To generate the heatmap, we evaluated sites of interest for subsequent clinical examination. For each video and static image, we fed them into the fully trained model and obtained the feature map of the final convolutional layer. Then, we calculated the heatmap by running Grad-CAM on this feature map. We mapped the resulting heatmaps to visualize the areas of the keyframe of videos that were most indicative of lesions of metastasis. As show in **Figure 3** the best TIN model focuses more on the location of the nodules than the other two models and the characteristics of the nodules are the most important basis for predicting whether the nodules will metastasize. The technology and details are presented in the **Supplementary Information** section.

### Statistical analysis

Differences between variable groups were analyzed using the Mann-Whitney U test. The chi-square test was used to compare rates between different groups. The performances of the three algorithms were evaluated by AUC, as well as the accuracy (ACC), sensitivity (SEN), specificity (SPE), positive predictive value (PPV), and negative predictive value (NPV). Intraobserver and interobserver agreements were compared using the Kappa value. All statistical analyses were two-sided, and *P*< 0.05 was considered statistically significant. We performed all statistical analyses using MedCalc (v.20.0.26; https://www.medcalc.org, Copyright MedCalc Software Ltd), Python software (v.3.8.5150.0; http://www.python.org), and SPSS software for Windows (v.20.0).

## Results

### Patient population and clinical characteristics

We studied a total of 421 breast lesions and finally enrolled 271 women with 271 malignant breast lesions for analysis. The average patient age was 52.90 years (range, 26–87 years) for the training and validation set and 53.31 years (range, 26–78 years) for testing set A. Among them, 9 (3.32%) patients had noninvasive carcinoma, 242 (89.3%) patients had invasive ductal carcinomas, and 9 (3.32%) had invasive lobular carcinomas. The mean tumor size was 0.23 cm (0.21-0.24 cm) for the training and validation set and 0.20 cm (0.18 cm-0.22 cm) for testing set A. Of the 271 patients, 133 (49.08%) had T1-stage tumors, and 138 (50.92%) had T2-stage tumors. The ALN of 116 (42.80%) patients was metastasized, and 155 (57.20%) patients had no lymph node metastasis. A detailed summary of patient demographics and tumor characteristics is provided in **Table 1**.

**Table 1:** Demographic Data for 271 Patients.

Characteristics	Total	Training and Validation Sets	Testing set A	P-value
Total patients	271	210 (77.5%)	61 (22.5%)	
Age				0.321
< 50 years old	88 (32.47%)	65 (30.95%)	23 (37.70%)	
≥ 50 years old	183 (67.53%)	145 (69.05%)	38 (62.30%)	
Tumor size (cm)	0.22	0.23	0.20	0.540
	(0.21-0.23)	(0.21-0.24)	(0.18-0.22)	
ALN of images				0.794
No lymph node metastasis	155 (57.20%)	121 (57.62%)	34 (55.74%)	
Lymph node metastasis	116 (42.80%)	89 (42.38%)	27 (44.26%)	
Clinical stage				0.548
T1 (≤ 20 mm)	133 (49.08%)	101 (53.46%)	32 (45.90%)	
T2 (≥ 21 mm)	138 (50.92%)	109 (47.54%)	29 (54.10%)	
Tumor location				
Central	4 (1.48%)	3 (1.43%)	1 (1.64%)	0.739
Inner upper quadrant	91 (33.58%)	73 (34.76%)	18 (29.51%)	
Outer upper quadrant	100 (36.90%)	73 (34.76%)	27 (44.26%)	
Inner lower quadrant	28 (10.33%)	23 (10.95%)	5 (8.20%)	
Outer lower quadrant	48 (17.71%)	38 (18.10%)	10 (16.39%)	
BI-RADS category				0.457
4A	43 (15.87%)	31 (15.30%)	12 (19.67%)	
4B	67 (24.72%)	49 (21.86%)	18 (29.51%)	
4C	94 (34.69%)	77 (36.61%)	17 (27.87%)	
5	67 (24.72%)	53 (26.23%)	14 (22.95%)	
Tumor pathology				0.076
DCIS	4 (1.48%)	3 (1.43%)	1 (1.64%)	
LCIS	2 (0.74%)	1 (0.48%)	1 (1.64%)	
Invasive ductal	242 (89.30%)	189 (90.00%)	53 (86.88%)	
Invasive lobular	9 (3.32%)	4 (1.90%)	5 (8.20%)	
Other pathology types	14 (5.16%)	13 (6.19%)	1 (1.64%)	

DCIS, ductal carcinoma in situ; LCIS, Lobular carcinoma in situ.

### DL model-based US videos showed better performance than DL model-based static images

**Figure 4:**
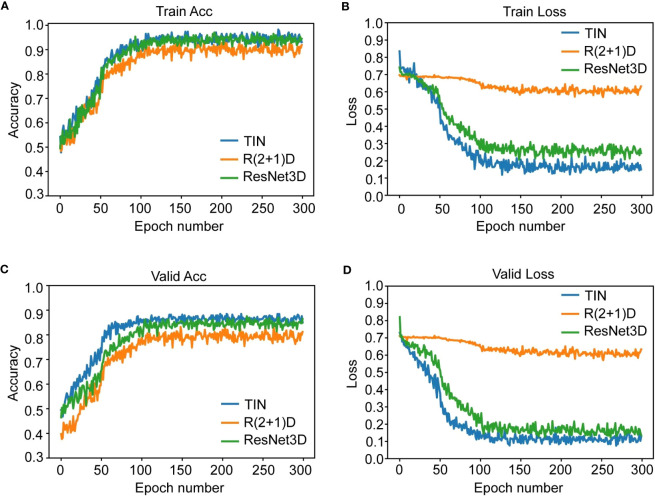
The figure shows the change curve of the loss function during the training of the dynamic video model. Subfigure **(A, B)** are Acc and loss curve for 300 training epoches. Subfigure **(C, D)** are Acc and loss curve for 300 validating epoches. TIN and ResNet converged well and stabilized at a small value of 0.2-0.3 in 300 training rounds. R2 + 1D converges relatively, but the trend is not obvious enough and the convergence value is about 0.6, which is a big gap. This also explains why its results on the test set are significantly lower than those of the other two models.

**Figure 5:**
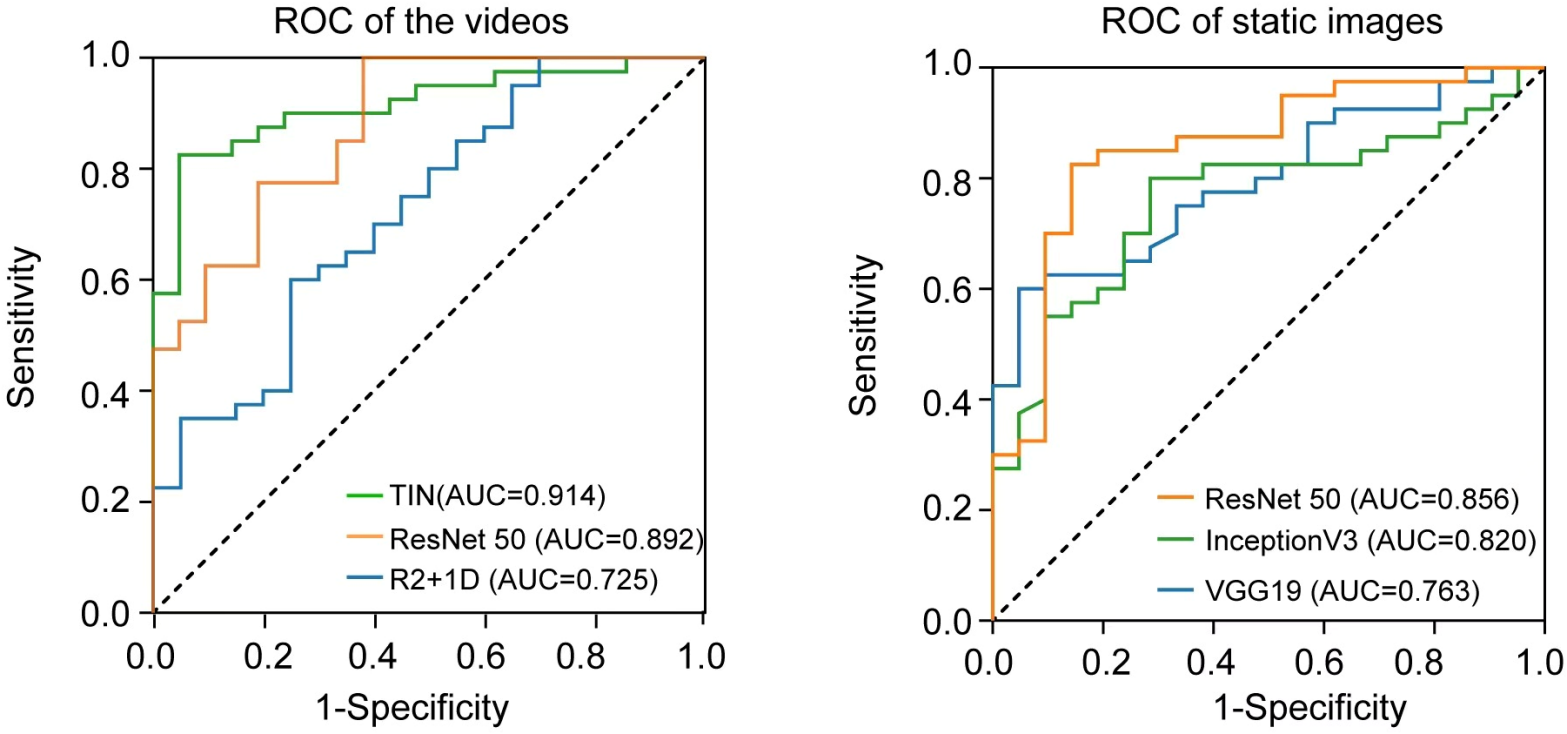
Comparison of receiver operating characteristic (ROC) curves between two models (video vs. static image) for predicting ALN metastasis.

We used three deep learning networks, TIN, R2 + 1D, and ResNet3D, to build the prediction model based on US videos. The change curve of the loss function during the training of the dynamic video model is shown in labels "A-D" of **Figure 4**. Additionally, we also built the prediction model based on US static images using ResNet50, InceptionV3, and VGG19 networks. Among the three US video DL models, the TIN network showed the best performance, with an accuracy, sensitivity, and specificity of 85.25% (52/61), 76.19% (16/21), and 90.00% (36/40), respectively. The AUC of the TIN model was better than that of the ResNet50 model (0.914, 95% CI: 0.843-0.985 vs. 0.856, 95% CI: 0.753-0.959, *P*< 0.05), as shown in **Figure 5**. The ResNet50 network showed the best performance among the US static image DL models, with an accuracy, sensitivity, and specificity of 80.33% (49/61), 66.67% (14/21), and 87.50% (35/40), respectively. The ResNet network is widely recognized for its extensive adoption as a classical model, primarily due to its incorporation of residual connections. These connections play a vital role in facilitating the smooth propagation of gradients and enhancing information flow across deeper layers. Consequently, this effectively addresses the issue of vanishing gradients. By leveraging this advantage, ResNet50 is capable of training deeper networks while maintaining performance levels, thereby capturing intricate features and achieving enhanced accuracy. In general the training of static images ignores the temporal correlation information between image frames, which is crucial for the prediction of whether the lymph node is metastatic or not, resulting in worse training results for the static image model. Detailed performance of the US video DL model and US static image model are provided in **Table 2**.

**Table 2:** The prediction of ALN status results (US video vs. US static image).

Image type	Model	Testing set A (N=61)						
		ACC%	SEN%	SPE%	PPV%	NPV%	F1-Score	Kappa
US video	TIN	85.25 (52/61)	76.19 (16/21)	90.00 (36/40)	80.00 (16/20)	87.8 (36/41)	0.7805	0.669
	R2 + 1D	63.93 (39/61)	66.67 (14/21)	62.50 (25/40)	48.28 (14/19)	78.12 (25/32)	0.5600	0.627
	ResNet3D	83.61 (51/61)	61.90 (13/21)	95.00 (38/40)	86.67 (13/15)	82.61 (38/46)	0.7222	0.610
US static image	ResNet50	80.33 (49/61)	66.67 (14/21)	87.50 (35/40)	73.68 (14/19)	83.33 (35/42)	0.7000	0.554
	InceptionV3	72.13 (44/61)	66.67 (14/21)	75.00 (30/40)	58.33 (14/24)	81.08 (30/37)	0.6220	0.403
	VGG19	65.57 (40/61)	85.71 (18/21)	60.05 (22/40)	50.00 (18/36)	88.00 (22/25)	0.6320	0.348

US video models of R2 + 1D, TIN and ResNet-3D architectures, US static image models of ResNet50, InceptionV3 and VGG19 architectures.

ACC, accuracy; SEN, sensitivity, SPE, specificity; PPV, positive predictive value; NPV, negative predictive value.

### DL model-based US videos achieved equal performance compared with ultra-sonographers

**Figure 6:**
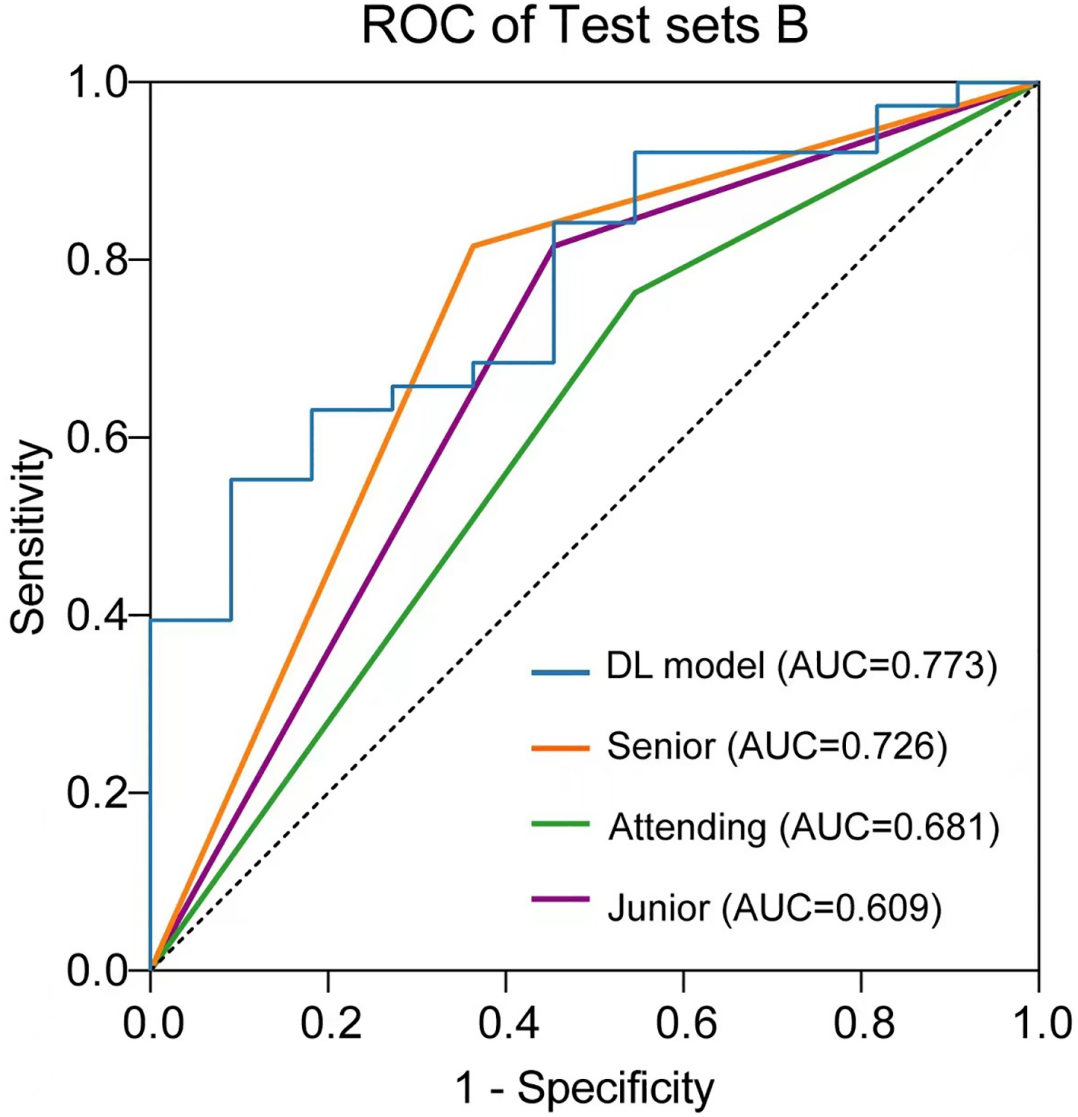
Comparison of receiver operating characteristic (ROC) curves between the ultra-sonographers and DL model.

The performance of the US video DL model (TIN) in predicting lymph node metastasis was compared with that of three breasts ultra-sonographers with varying levels of experience using the pathological reference standard. For independent testing set B, consisting of 49 lesions, the senior ultrasonographer had an accuracy of 79.59% (39/49), the sensitivity of 63.64% (7/11), and specificity of 84.21% (32/38), while the DL model had an accuracy of 83.67% (41/49), the sensitivity of 54.55% (6/11), and specificity of 92.11% (35/38). As shown in **Table 3**, the ROC curves for the DL model and the ultra-sonographers, as seen in **Figure 6**, demonstrated that the ultra-sonographers achieved AUCs of 0.609 to 0.726 based on image classification alone, while the AUC of the DL model (TIN) was 0.773 (95% CI: 0.630-0.915, P<0.05), which was equal to or slightly higher than that of the ultra-sonographers with different levels of experience. However, there was no statistically significant difference between the DL model and ultra-sonographers. The external test set exhibits a distinct data distribution from the original test set, while test set B has a smaller data volume in comparison to the initial test set. As a result, there is a slight decrease in performance when evaluating test set B as compared to test set A.

**Table 3:** The performance of the DL models for predicting lymph node metastasis compared with the ultrasonographers.

Finding	A (ALN)	B (ALN)	C (ALN)	DL model (breast)
ACC (%)	67.35 (33/49)	73.47 (36/49)	79.59 (39/49)	83.67 (41/49)
SEN (%)	45.45 (5/11)	54.55 (6/11)	63.64 (7/11)	54.55 (6/11)
SPE (%)	73.68 (28/38)	78.94 (30/38)	84.21 (32/38)	92.11 (35/38)
PPV (%)	33.33 (5/15)	42.86 (6/14)	53.85 (7/13)	66.67 (6/9)
NPV (%)	82.35 (28/34)	85.71 (30/35)	88.89 (32/36)	87.50 (35/40)
Kappa	0.169 ± 0.148	0.305 ± 0.150	0.449 ± 0.147	0.499 ± 0.153

A, Junior; B, Attending; C, Senior; ACC, accuracy; SEN, sensitivity; SPE, specificity; PPV, positive predictive value; NPV, negative predictive value.

### Model interpretation

**Figure 7:**
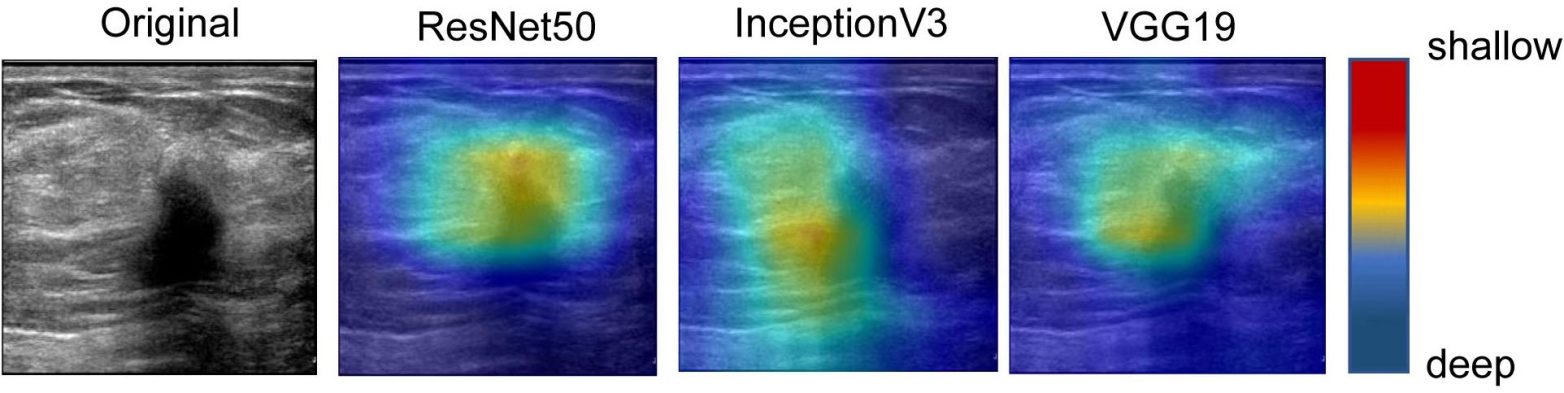
The visual activation diagram of the prediction results of the static image model. The brighter the place, the higher the model’s attention. It can be seen that all models basically pay attention to the characteristics of the nodule area, but they are not concentrated enough. Among them, Inception V3 and VGG19 models focus on the left side of the nodule, which also affects the final prediction results to some extent.

The heatmaps generated by the US video DL model and US static image model are shown in **Figure 7**. The green and blue backgrounds represent the low forecast range, while the darker the feature color, the higher the attention of the model. This indicates that the trained model accurately identifies the areas that contribute the most to the final prediction result, which is also confirmed by clinicians. The focus heatmap continues to highlight the key elements in the current image, demonstrating that the characteristics of malignant lesions have been well studied and serve as the basis for LNM classification. These heatmaps have great clinical value in guiding subsequent clinical examination and treatment planning.

## Discussion

For the past three decades, sentinel lymph node biopsy (SLNB) has been established as a standard of care for clinical node-negative breast cancer patients, allowing for the avoidance of axillary lymph node dissection (ALND) when SLNs are not involved with metastases (37). Moreover, ALND can also be omitted in selected patients with T1 or T2 breast cancer and fewer than two positive sentinel lymph nodes who undergo breast-conserving surgery (38). However, accurately predicting the metastatic status of ALN pre-operatively and/or intra-operatively remains challenging, despite numerous studies evaluating the risk of LN metastases. Therefore, there is a critical need to accurately predict the metastatic status of ALNs in a non-invasive manner. In this study, we aimed to utilize DL models with US video images in combination with clinicopathological factors to achieve the goal of predicting lymph node metastasis.

US has shown great promise in the assessment of ALN status in patients with early breast cancer, however, with the features of unclear margins, irregular shapes, or loss of fatty hilum, only visualized nodes can be analyzed and most of negative ALN with micro metastasis or without suspicious imaging features are missed (5). The accurate detection of lymph node micro metastasis is essential for guiding surgical decision making, and adjuvant therapy. Recent advances in AI models, such as DL technology, have been applied to breast US imaging (18, 39), and some studies have reported the effectiveness of DL models using a convolutional neural network (CNN) for the prediction of clinical ALN metastasis, with AUCs ranging from 0.72 to 0.89 (23, 40). However, these studies were based solely on ultrasound static (single frame) images, which may have resulted in the loss of many subtle or important lesion features and even caused the neglect of small lesions, as reported previously. In contrast, our study analyzed US videos containing multiple frames of images from the same patient while also considering the correlation between different frames. For video processing, additional temporal convolutions are employed. These convolutions help capture motions and temporal dependencies across consecutive frames. They typically involve 3D convolutions, where filters have both spatial and temporal dimensions. These additional operations enable deep learning models to capture temporal patterns, motion information, and long-term dependencies (41, 42). By combining the features between frames, the model to capture temporal relationships, dependencies, and patterns that may not be apparent when considering individual frames in isolation. As a result, the inclusion of temporal information through feature fusion enhances the overall performance of video-based tasks.

We demonstrated that our DL model achieved better performance than static images previously studied, resulting in higher accuracy (85.25% vs. 80.33%) and specificity (90.00% vs. 87.50%). Furthermore, compared to senior ultra-sonographers in testing set B, the DL model also showed higher accuracy (83.67% vs. 79.59%) and specificity (92.11% vs. 84.21%). These significant improvements suggest that this model may be useful in a clinical setting for predicting lymph node metastasis, potentially assisting ultra-sonographers in making decisions regarding appropriate axillary rescanning. This DL model might have great potential to serve as a noninvasively imaging biomarker to replace SLND and/or ALND for patients with early-stage breast cancer. Especially, the model could assist breast surgeons to make clinical decisions for appropriate axillary management, e.g., omission of SLN biopsy in the node negative patients.

To the best of our knowledge, our study is the first to demonstrate the effectiveness of DL model in predicting ALN metastases using ultrasound (US) video images. While previous studies have used deep learning techniques for lymph node classification, they mainly focused on magnetic resonance imaging (MRI) images and CT (40, 43). Yu et al (44). developed an effective preoperative MRI radiomics evaluation methodology for ALN status in patients with early-stage invasive breast cancer, utilizing machine learning methods. A multiomic signature, which combined tumor and lymph node MRI radiomics, clinical and pathologic traits, and molecular subtypes, exhibited superior ALN status prediction performance, with AUCs of 0.90, 0.91, and 0.93 across the training, external validation, and prospective-retrospective validation cohorts respectively. Zhang et al (45), on the other hand, established and verified a multiparametric MRI-based radiomics nomogram for predicting axillary sentinel lymph node (SLN) burden in early-stage breast cancer before treatment. The developed radiomics nomogram, which integrated radiomics signature and MRI-determined ALN burden, demonstrated good calibration in predicting SLN burden, with AUCs of 0.82, 0.81, and 0.81 across the training, validation, and test cohorts respectively. Wu et al (46). formulated and assessed non-invasive models leveraging contrast-enhanced spectral mammography (CESM) to estimate the risk of non-sentinel lymph node (NSLN) metastasis and axillary tumor burden among breast cancer patients with 1-2 positive sentinel lymph nodes (SLNs). The radiomics nomogram for NSLN metastasis status prediction yielded an AUC of 0.85 in the test set and 0.82 in the temporal validation set. Yang et al (46). created a deep learning signature based on staging CT to preoperatively predict sentinel lymph node metastasis in breast cancer. The deep learning signature presented an impressive discriminative capability, with an AUC of 0.801 in the primary cohort and 0.817 in the validation cohort.

The use of US video images offers several advantages over MRI, such as cost-effectiveness, wider availability, and no need for contrast agent administration. Additionally, since the usual practice involves ultrasound-guided biopsy after MRI findings, our study further supports the potential of US images in developing robust DL algorithms. These algorithms have practical value in predicting lymph node status, assisting in decision-making, and improving patient management.

Importantly, this DL model offers several advantages for evaluating the status of ALNs. First, it can provide a probability of ALN metastasis for input breast US video images, potentially preventing misdiagnosis of axillary malignant lymph nodes by junior ultra-sonographers with less than 5 years of experience. Second, the interpretability of the DL model is increased through the attention heatmap generated by CAM, which is a tool for visualizing CNN networks. The attention heatmap allows us to see which regions in the image were relevant to this class (47). Therefore, we can infer which keyframe of the input videos is focused on by the DL model using the attention heatmaps. Third, the DL model’s performance allows rescanning of patients with negative axillary lymph node ultrasound. The attention heatmap of the metastatic nodes represented evidence of DL model classification and could assist in clinical decision making by directly identifying the ROI.

However, this study had several limitations. First, the DL model was developed for predicting the probability of ALN metastasis in early breast cancer patients with lesion ultrasonic videos. Ultra-sonographers still need to identify lymph nodes on US and input the videos. Second, this was a retrospective study, and the dataset was limited. Some of the patients with negative lymph nodes may have positive lymph nodes if followed up for a long enough time. Third, this study was based on three centers, and more external validation studies are necessary.

In conclusion, based on US video images, we developed a prediction model of ALN metastasis in early breast cancer patients using deep learning approach and obtained

good performance in the independent external validation cohort. The model may provide an effective diagnostic reference for metastasis in early breast cancer patients clinical use.

## Data availability statement

The raw data supporting the conclusions of this article will be made available by the authors, without undue reservation.

## Ethics statement

The studies involving human participants were reviewed and approved by Xiang’an Hospital of Xiamen University. The patients/participants provided their written informed consent to participate in this study. Written informed consent was obtained from the individual(s) for the publication of any potentially identifiable images or data included in this article.

## Author contributions

W-BL and Z-CD contributed equally to this work and share first authorship. W-BL collected data. W-BL and Z-CD drafted the manuscript. QD participated the data processing. Y-JL, J-XG, J-GW performed the analysis. W-HH conceived the study. All authors contributed to the article and approved the submitted version.
