# Supplementary figures and images for: Prediction of axillary lymph node metastasis in early breast cancer patients with ultrasonic videos based deep learning
Source: Front Oncol. 2023 Sep 1;13:1219838. doi: 10.3389/fonc.2023.1219838 (PMC10503049; doi:10.3389/fonc.2023.1219838)

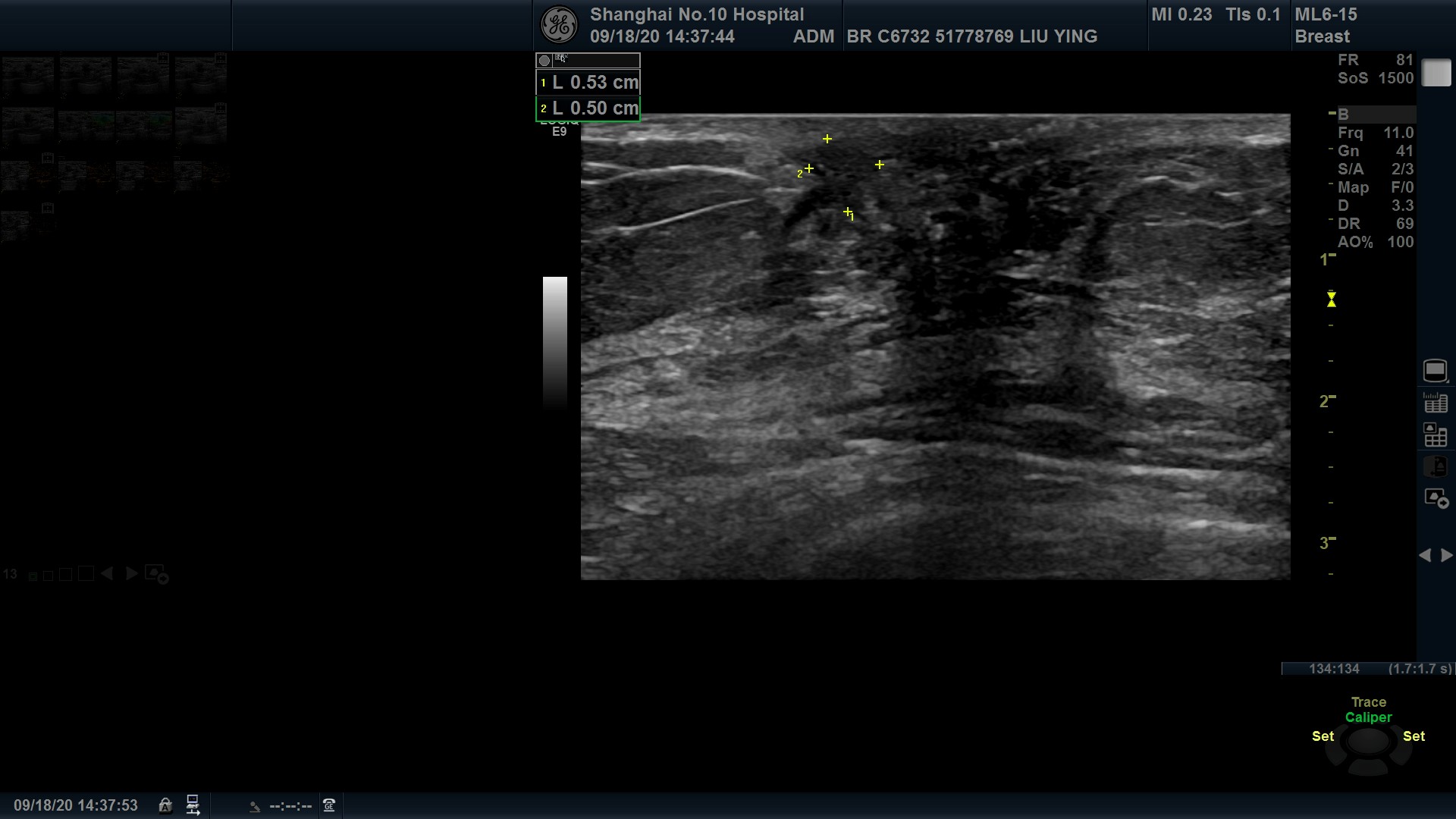

Supplement: Supplementary file 1 [file DataSheet_1.zip › 3/5612464_高永珍/高永珍_54949390.jpg]

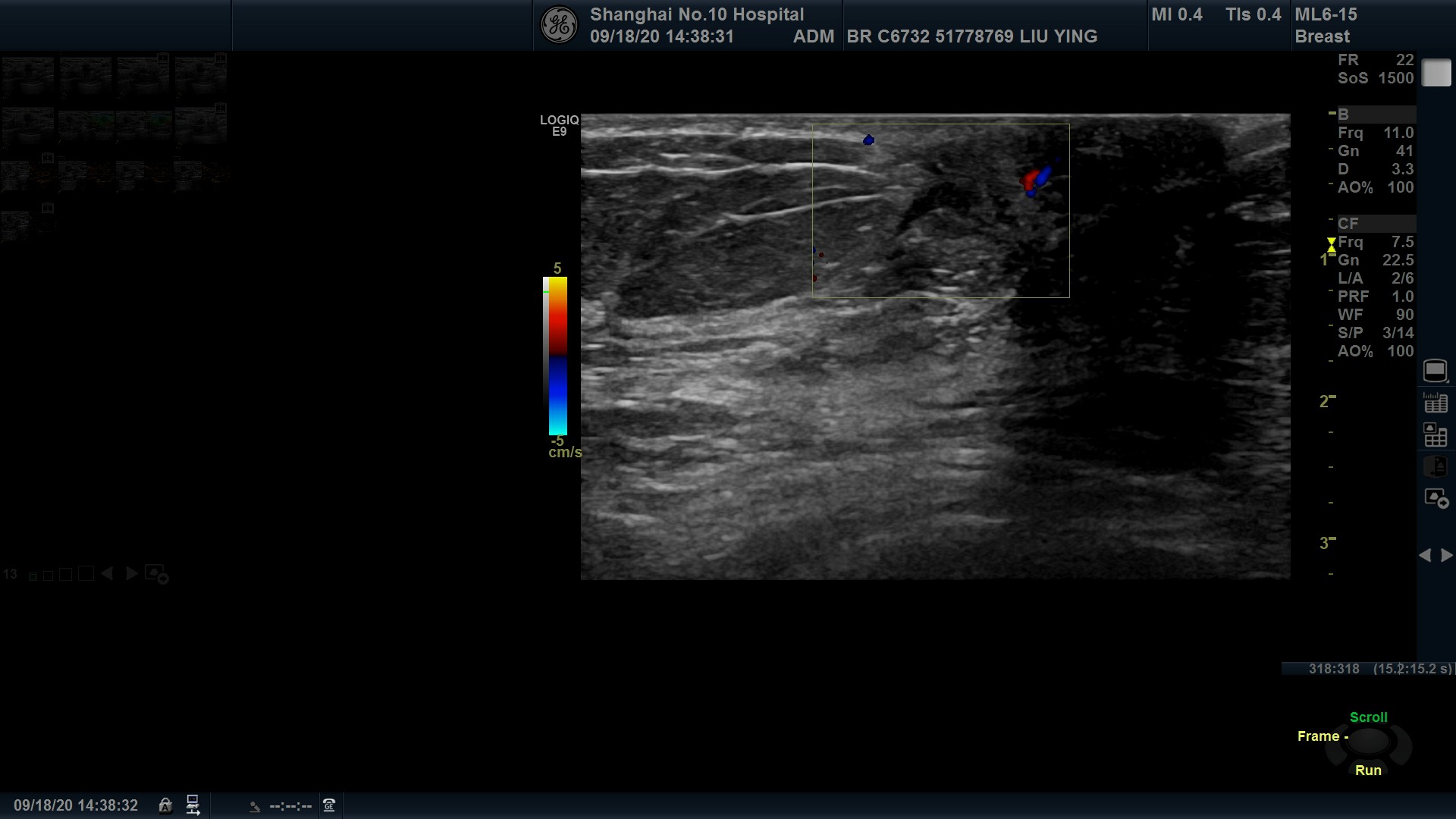

Supplement: Supplementary file 1 [file DataSheet_1.zip › 3/5612464_高永珍/高永珍_54949400.jpg]

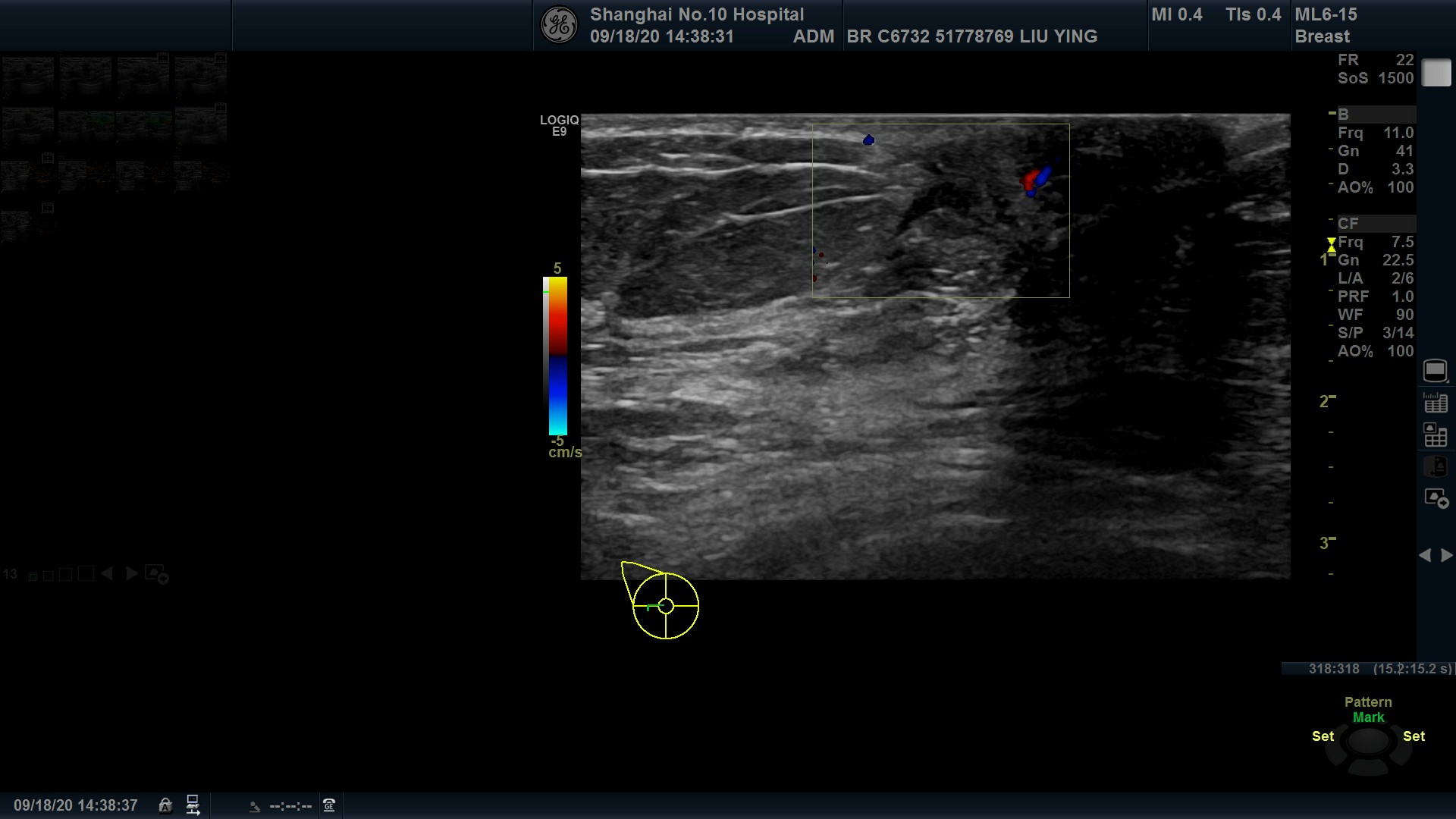

Supplement: Supplementary file 1 [file DataSheet_1.zip › 3/5612464_高永珍/高永珍_54949405.jpg]

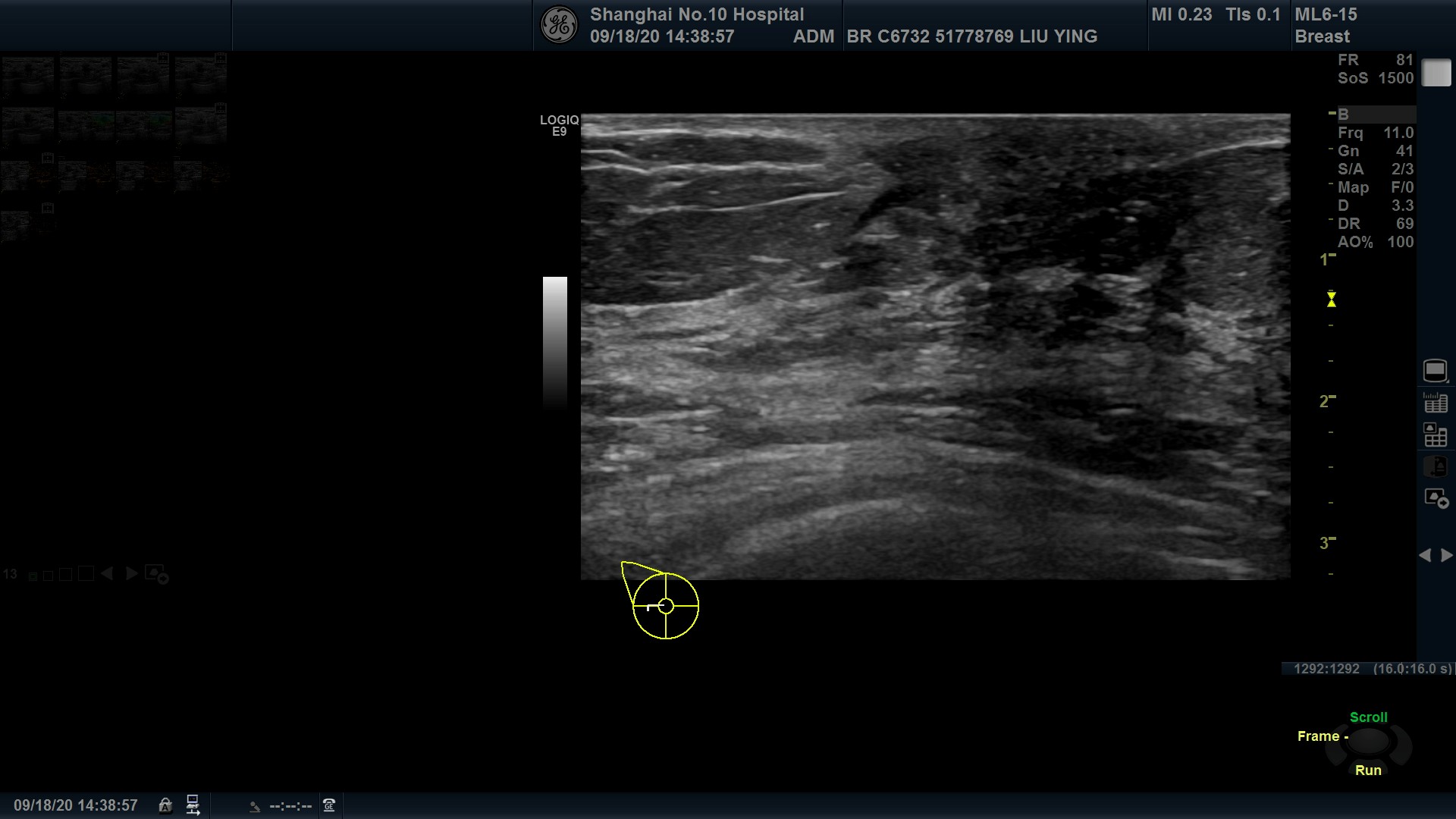

Supplement: Supplementary file 1 [file DataSheet_1.zip › 3/5612464_高永珍/高永珍_54949411.jpg]

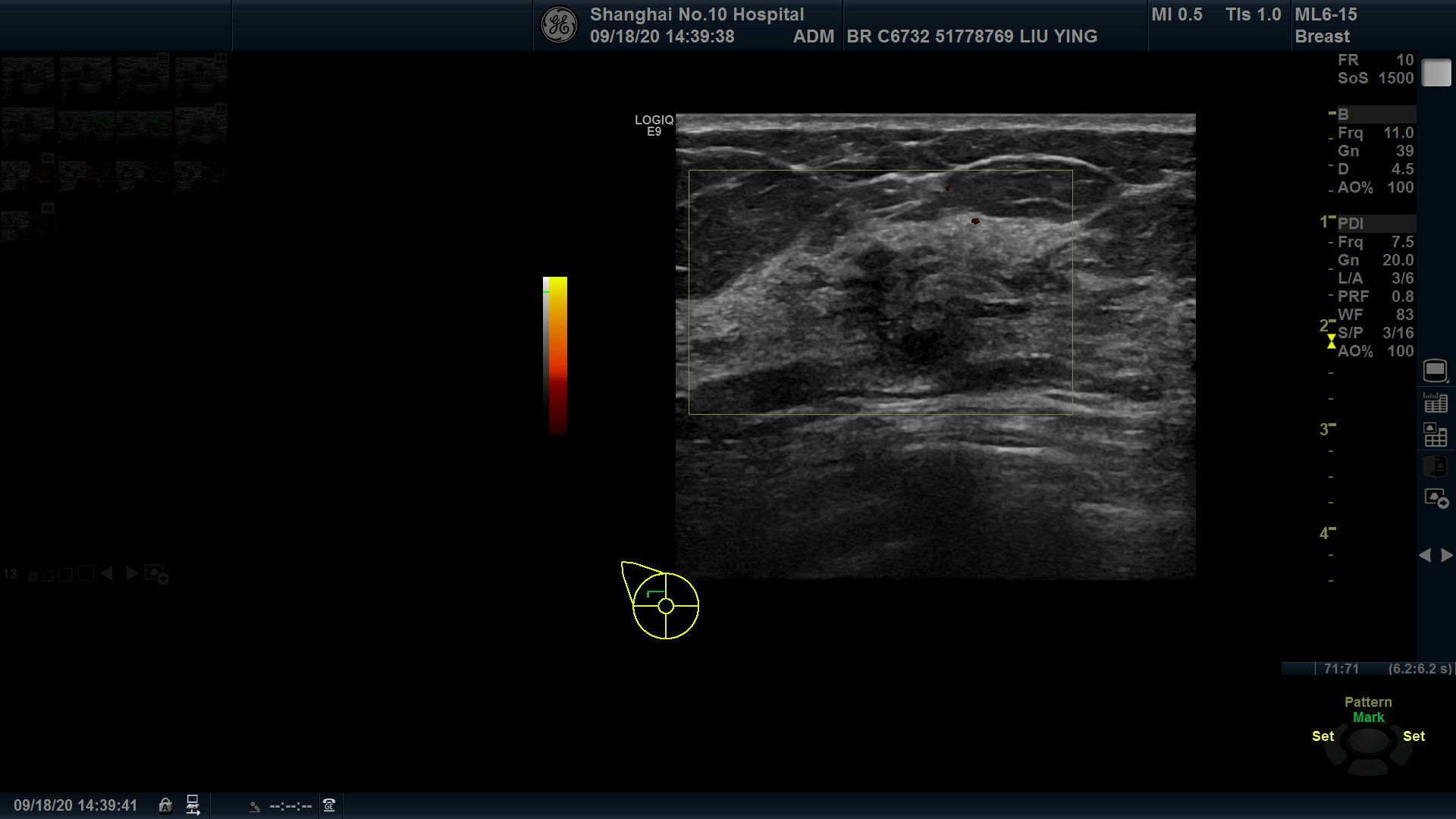

Supplement: Supplementary file 1 [file DataSheet_1.zip › 3/5612464_高永珍/高永珍_54949426.jpg]

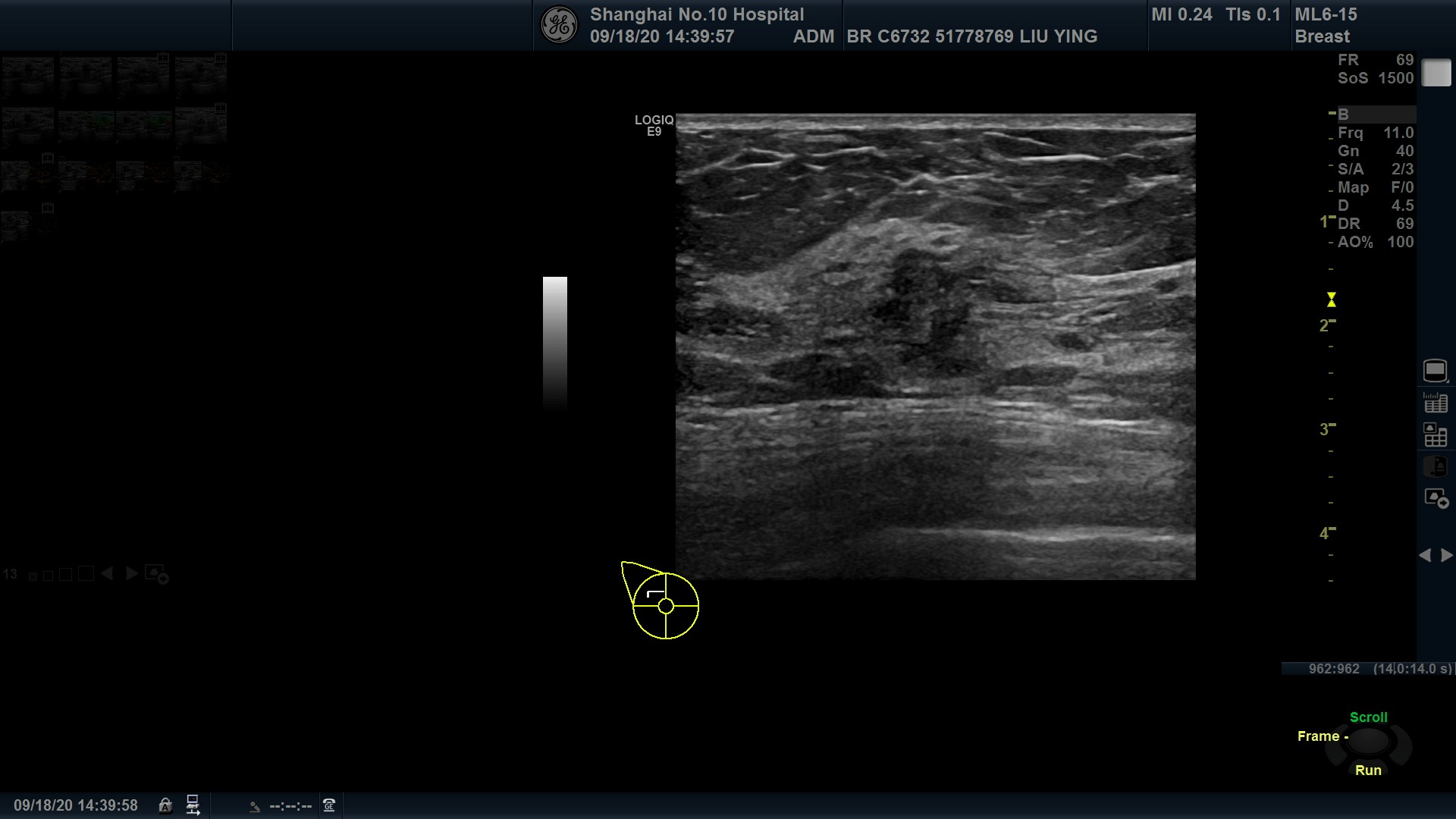

Supplement: Supplementary file 1 [file DataSheet_1.zip › 3/5612464_高永珍/高永珍_54949431.jpg]

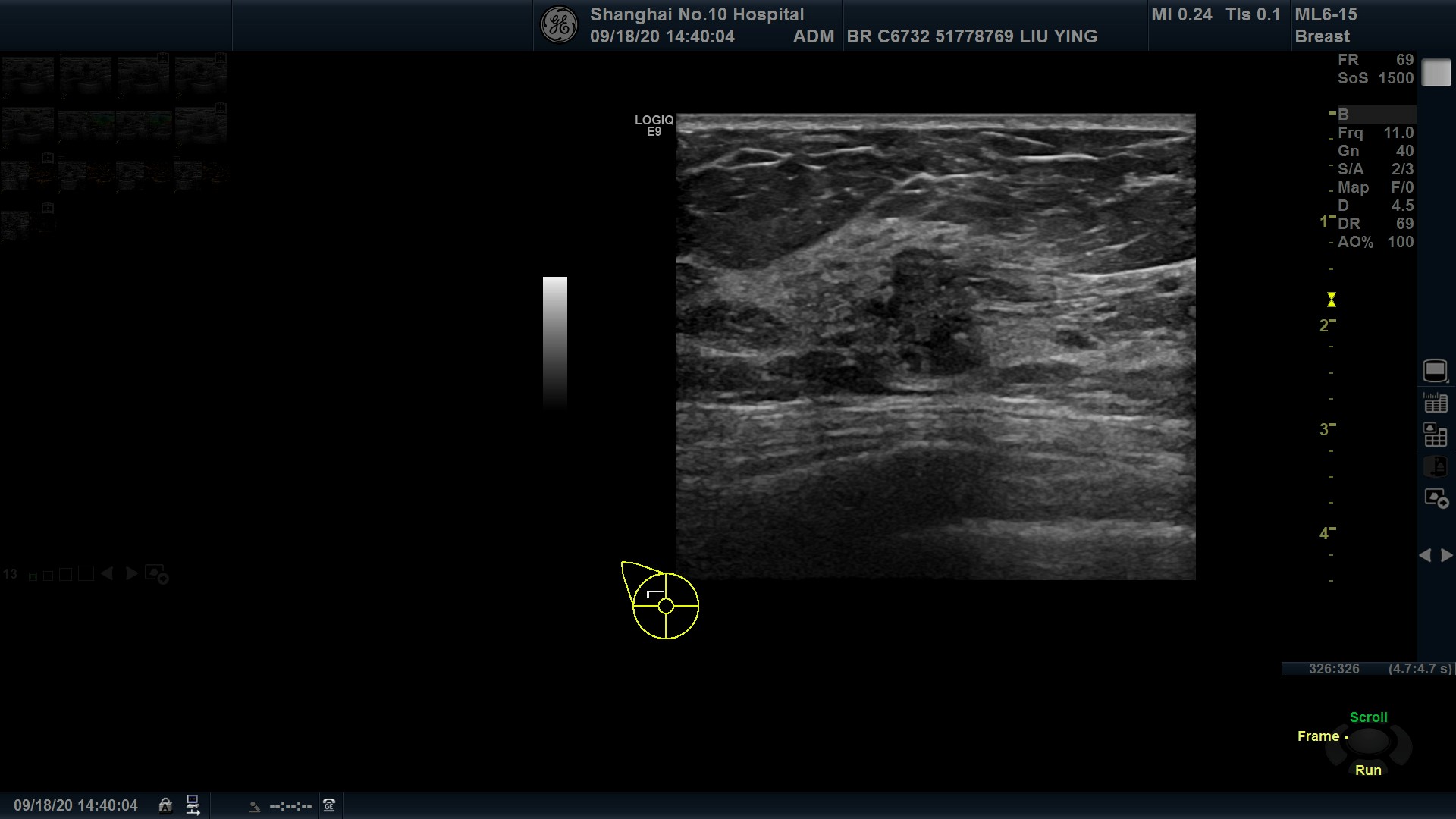

Supplement: Supplementary file 1 [file DataSheet_1.zip › 3/5612464_高永珍/高永珍_54949438.jpg]

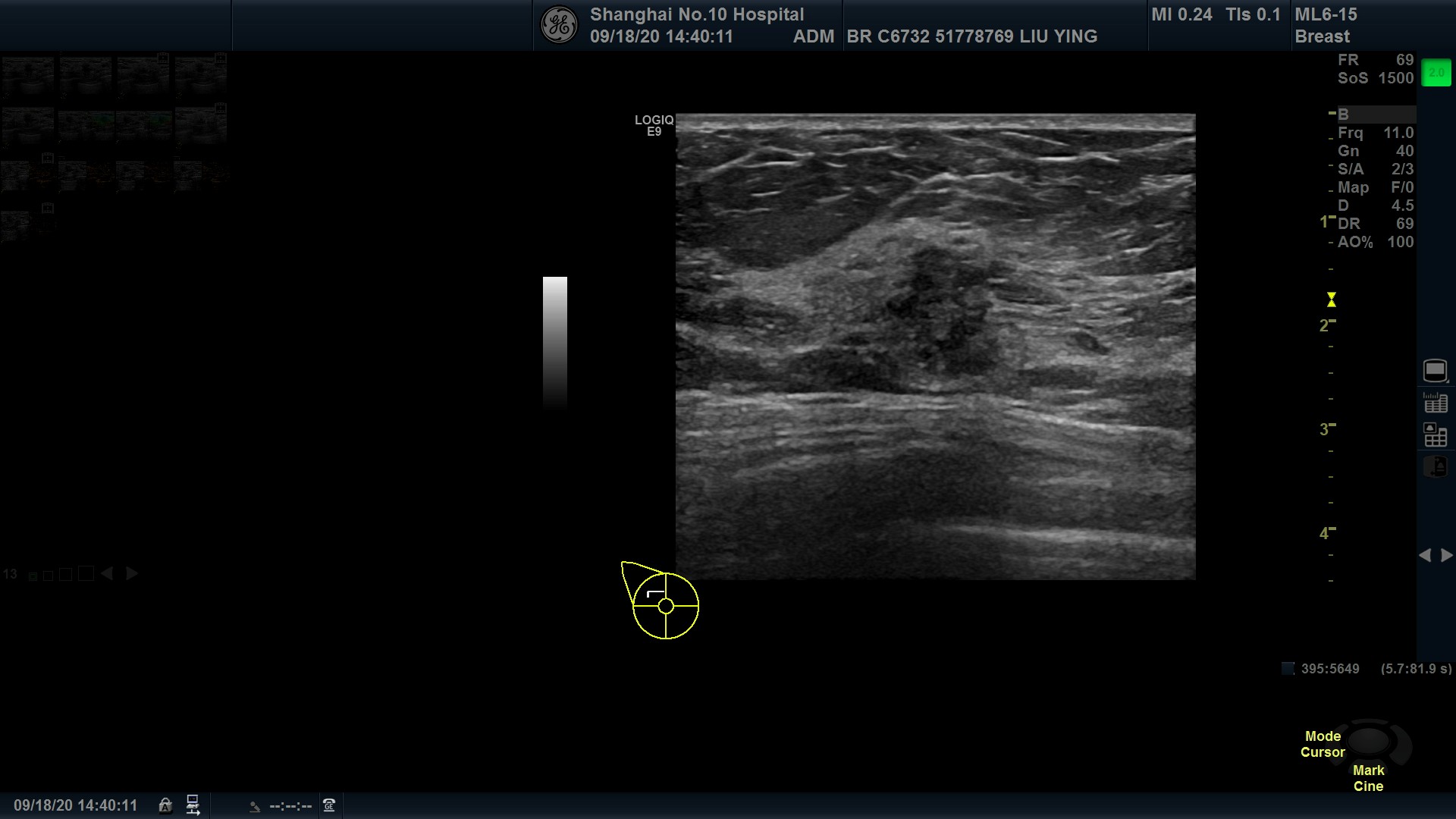

Supplement: Supplementary file 1 [file DataSheet_1.zip › 3/5612464_高永珍/高永珍_54949442.jpg]

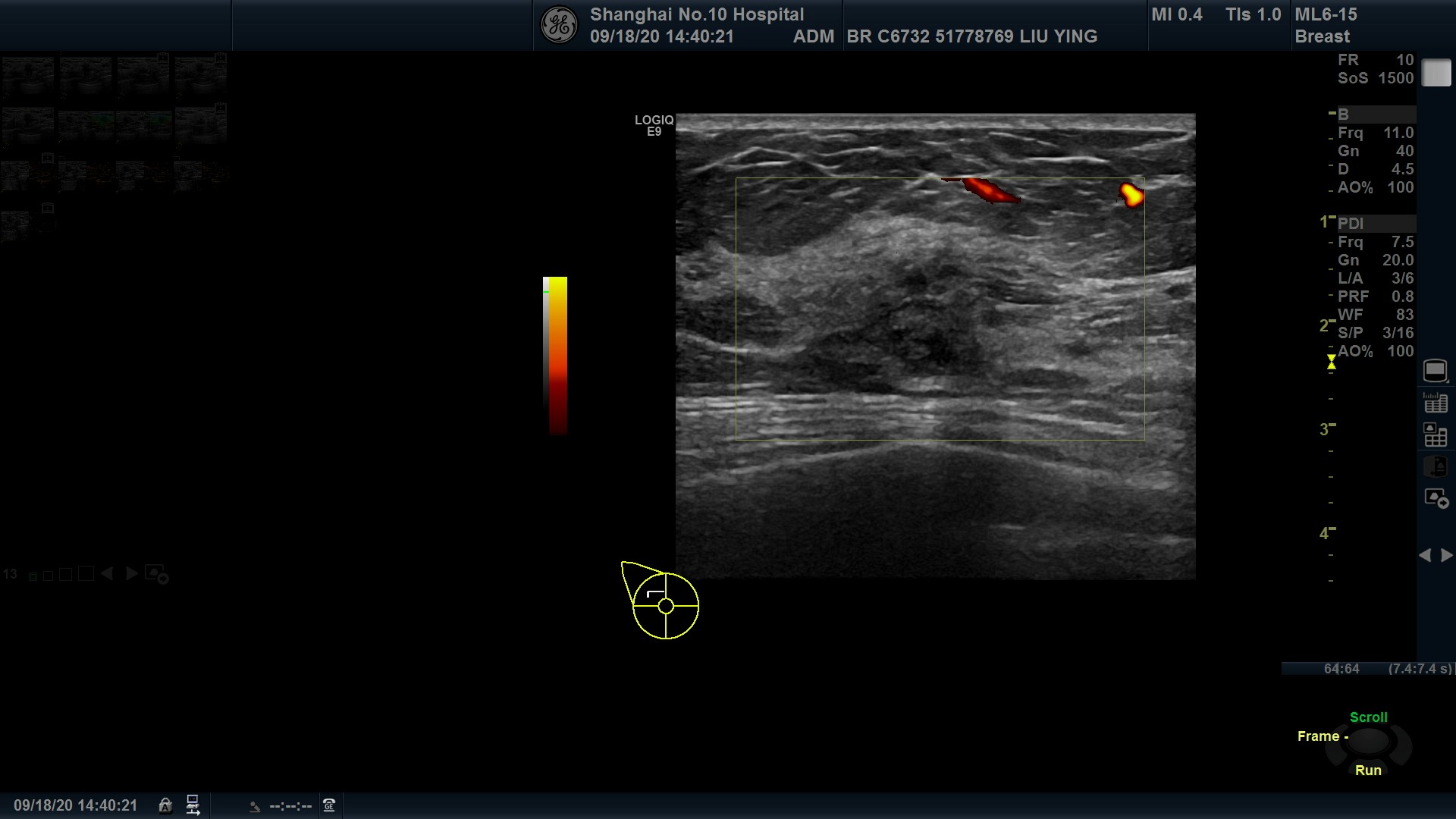

Supplement: Supplementary file 1 [file DataSheet_1.zip › 3/5612464_高永珍/高永珍_54949447.jpg]

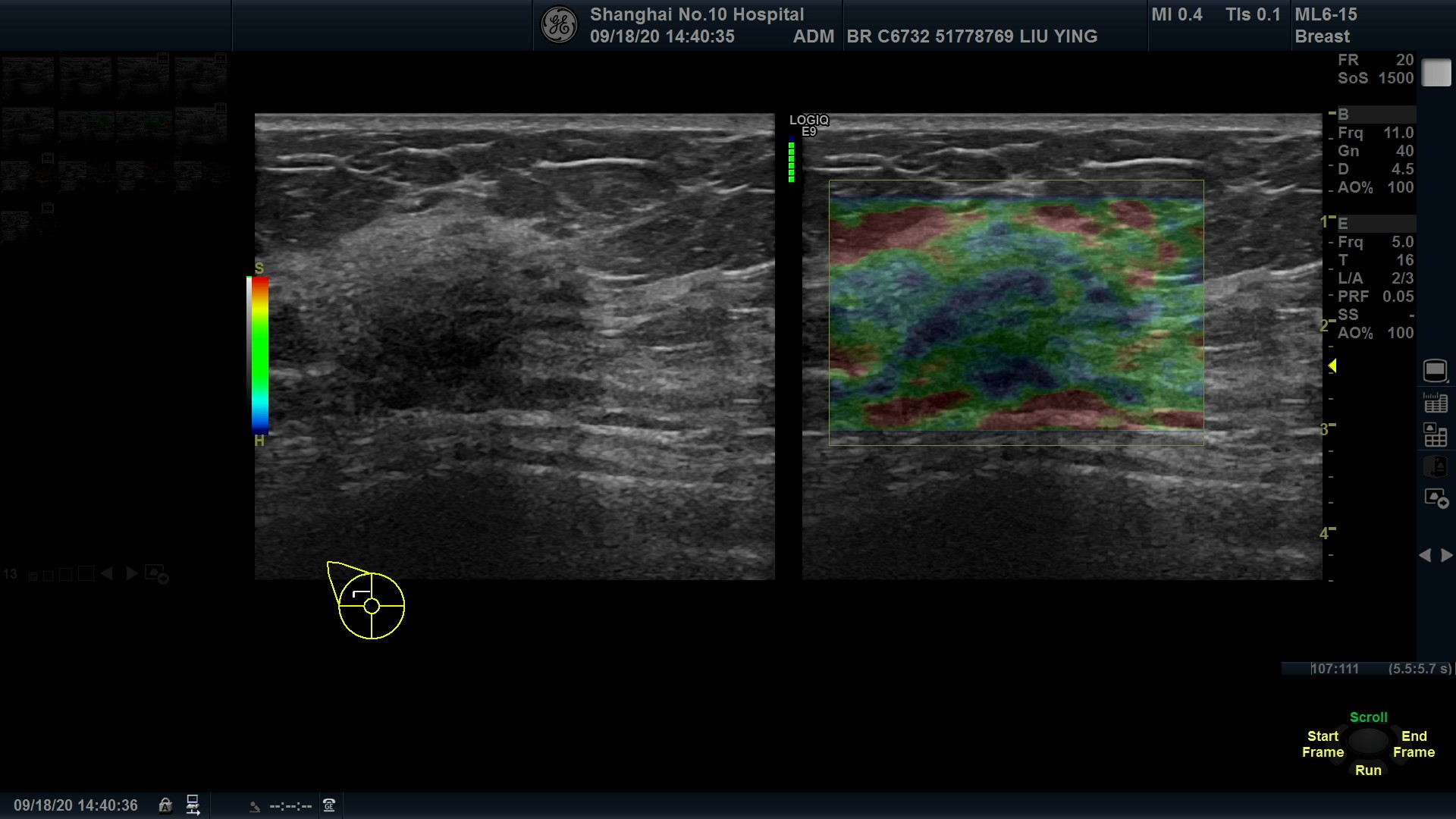

Supplement: Supplementary file 1 [file DataSheet_1.zip › 3/5612464_高永珍/高永珍_54949451.jpg]

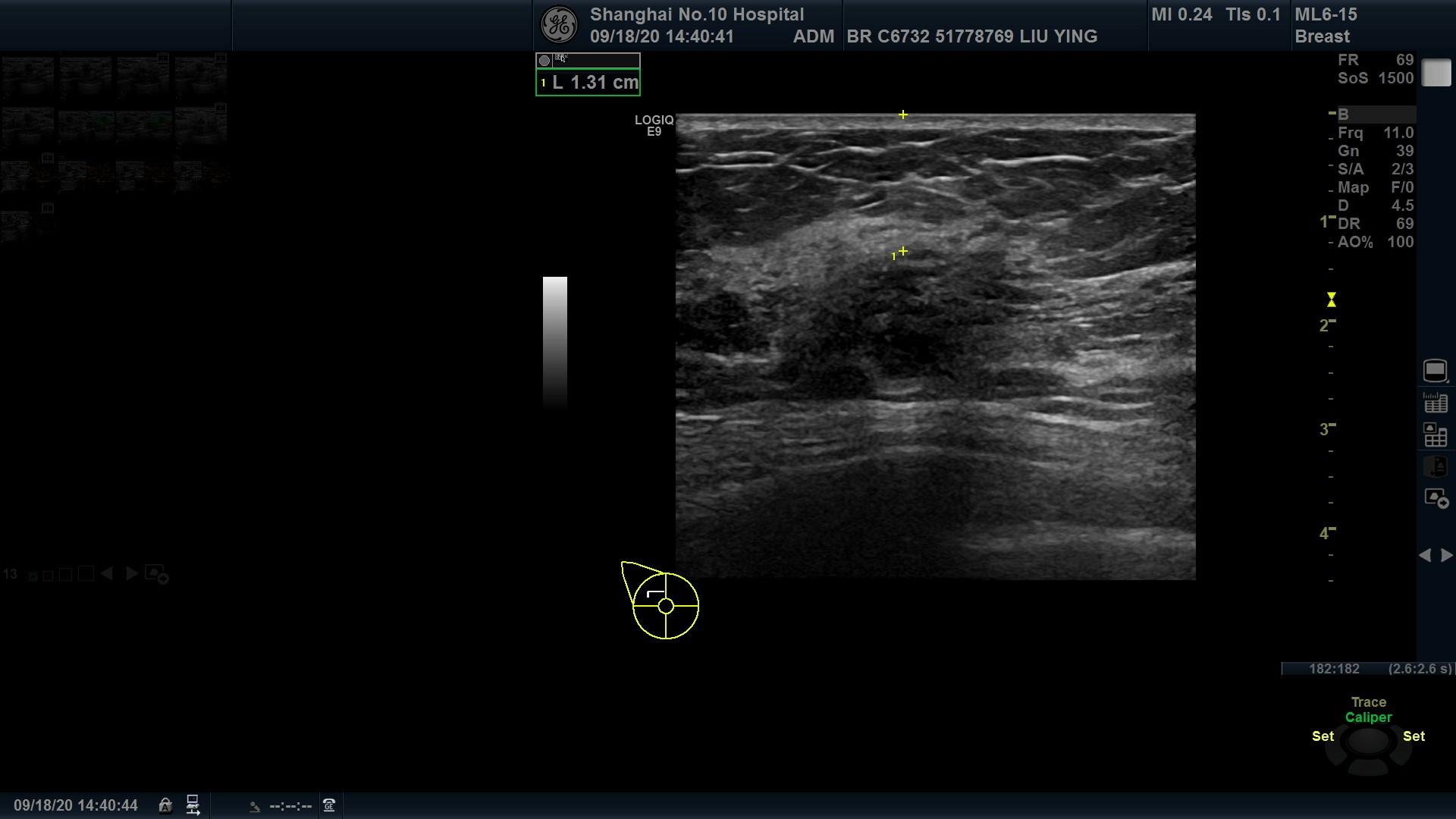

Supplement: Supplementary file 1 [file DataSheet_1.zip › 3/5612464_高永珍/高永珍_54949455.jpg]

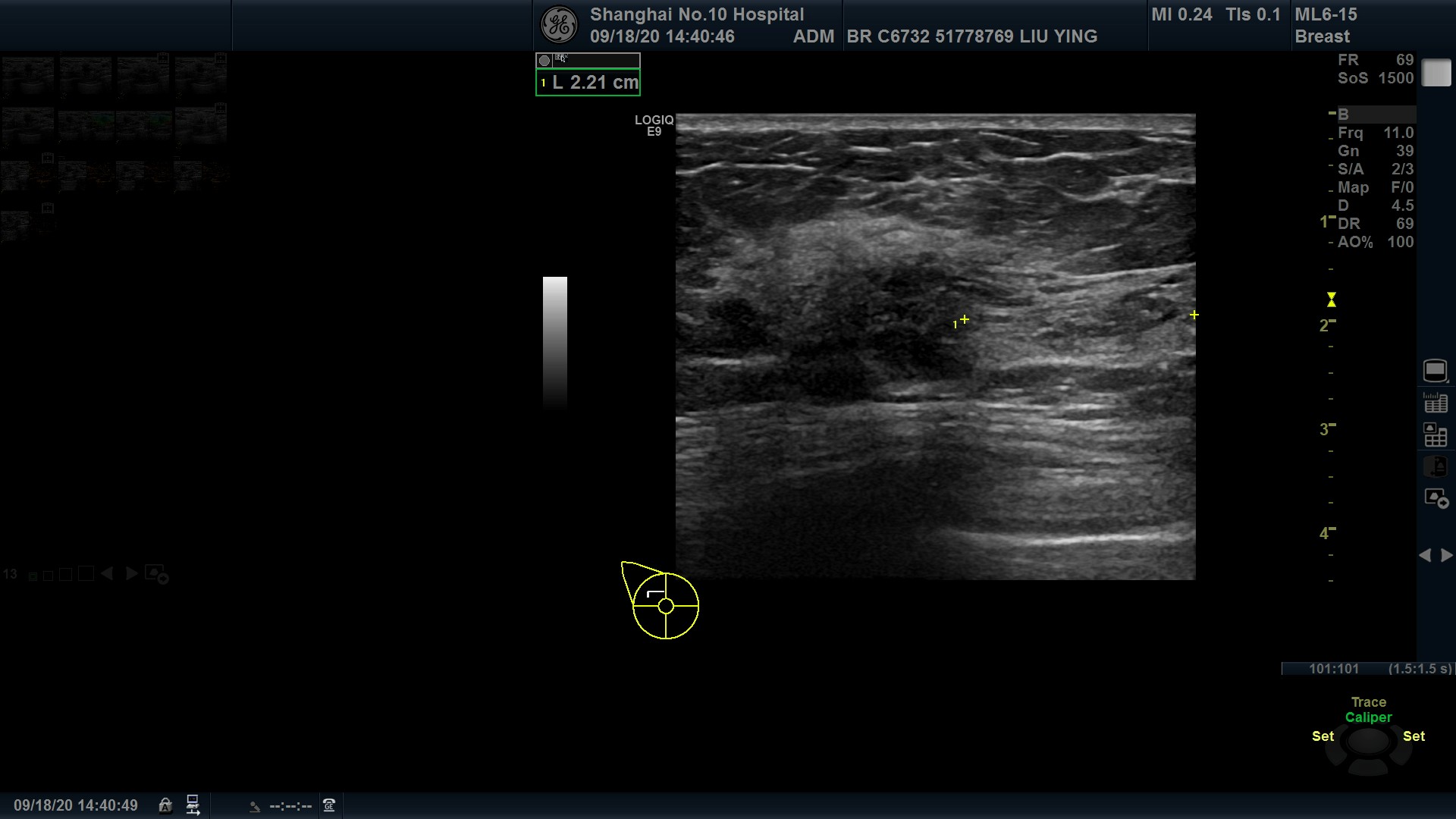

Supplement: Supplementary file 1 [file DataSheet_1.zip › 3/5612464_高永珍/高永珍_54949458.jpg]

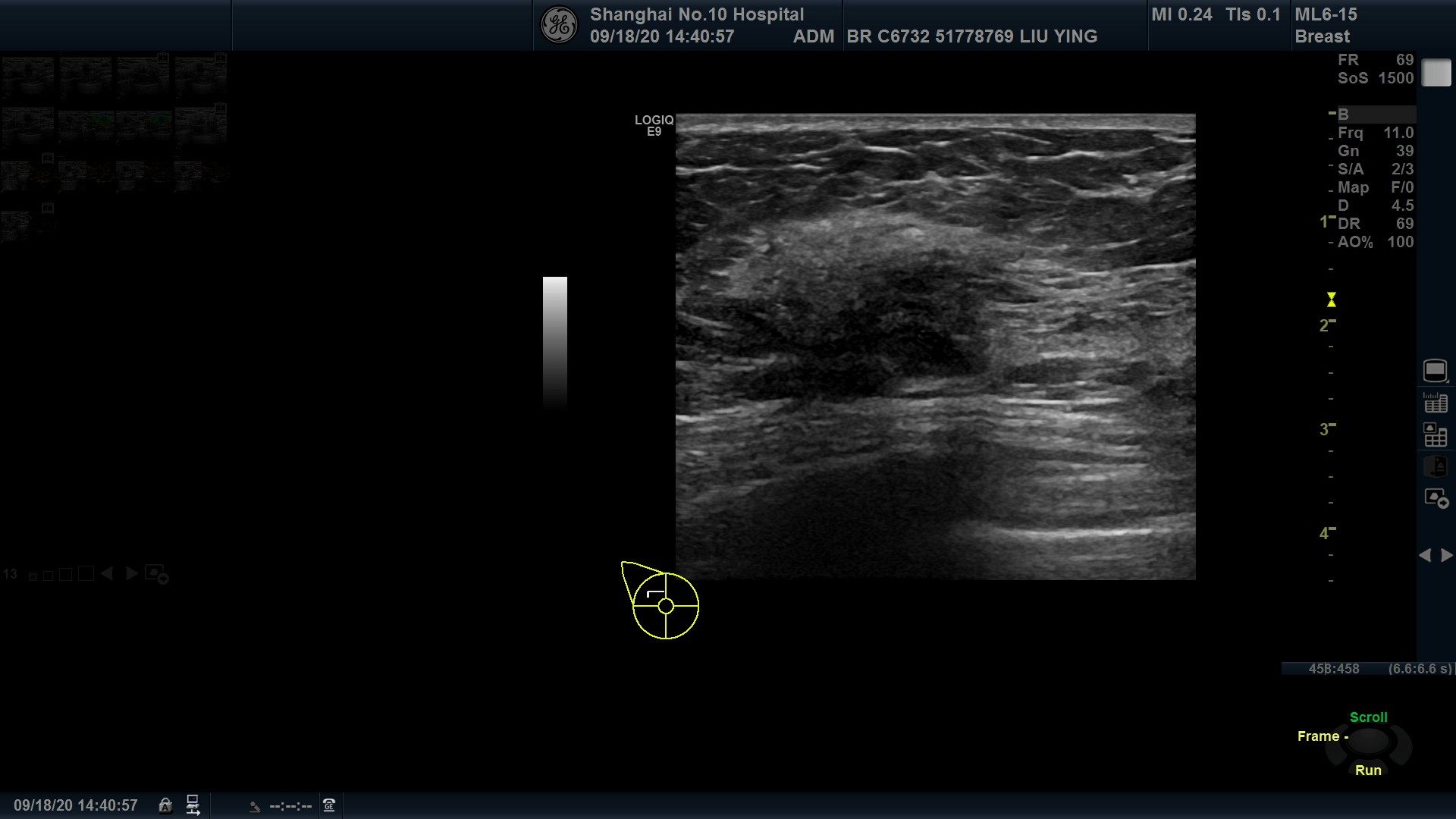

Supplement: Supplementary file 1 [file DataSheet_1.zip › 3/5612464_高永珍/高永珍_54949461.jpg]

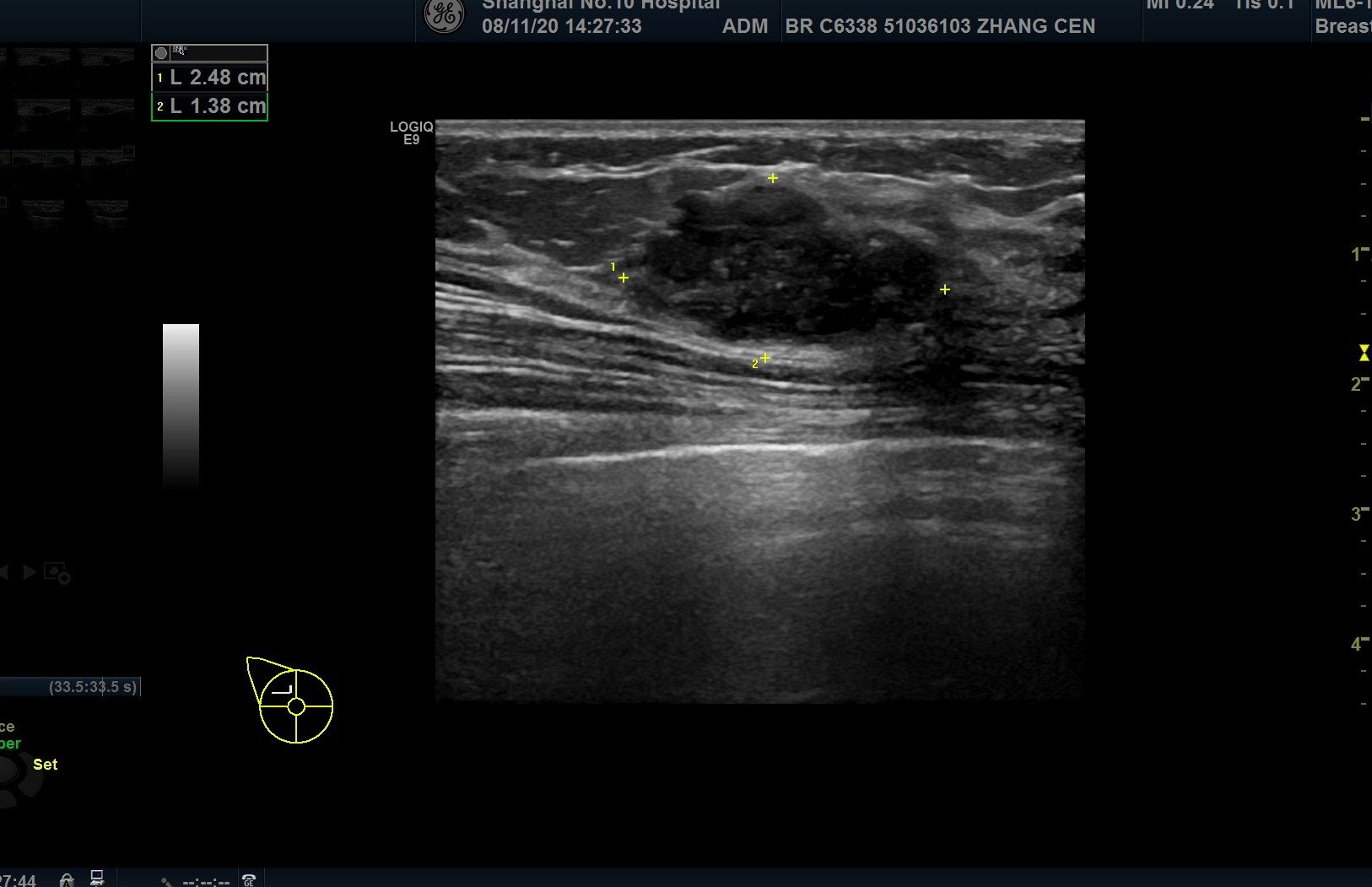

Supplement: Supplementary file 1 [file DataSheet_1.zip › 3/700168_杨柳依/杨柳依_44703244.jpg]

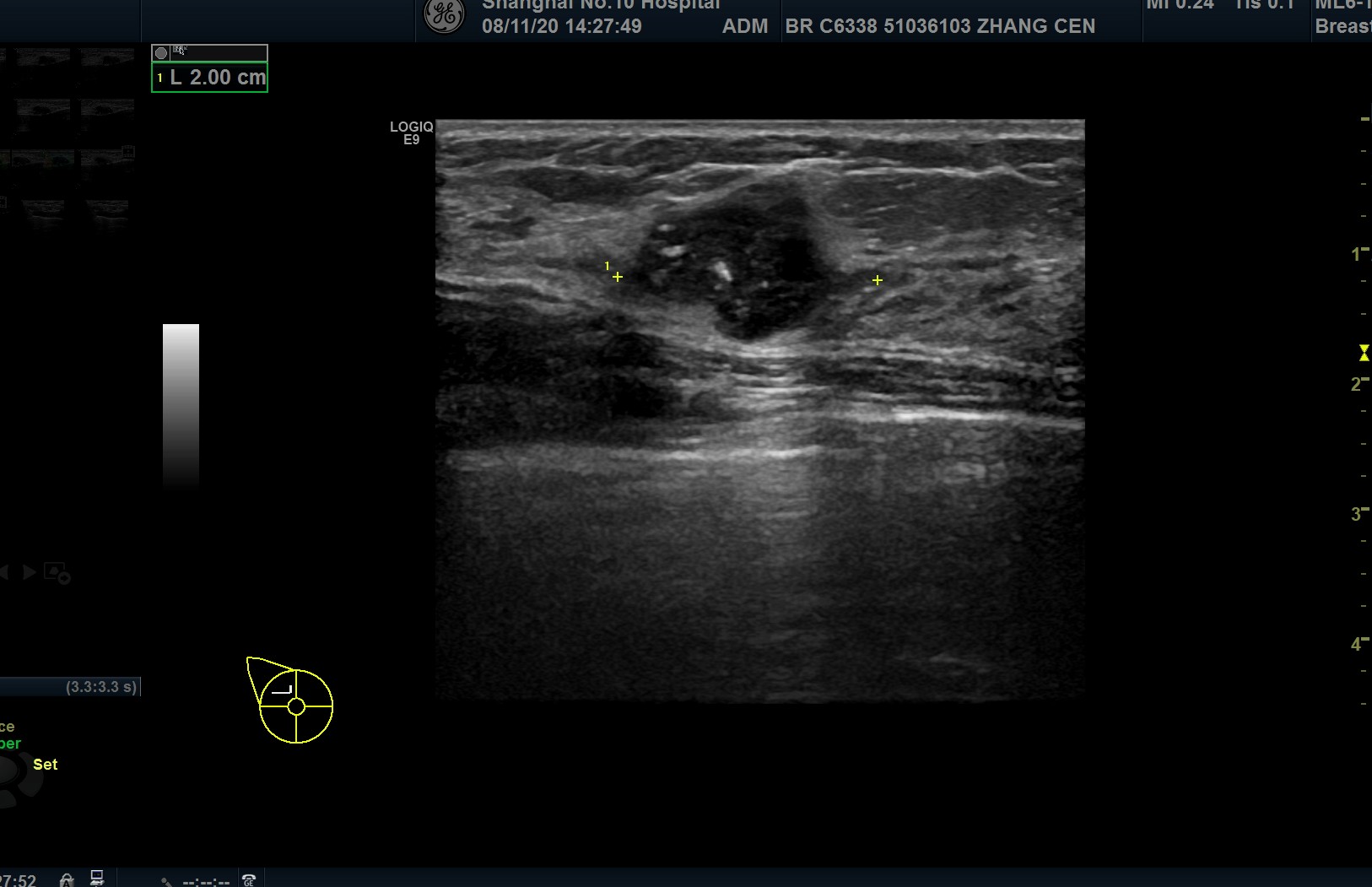

Supplement: Supplementary file 1 [file DataSheet_1.zip › 3/700168_杨柳依/杨柳依_44703248.jpg]

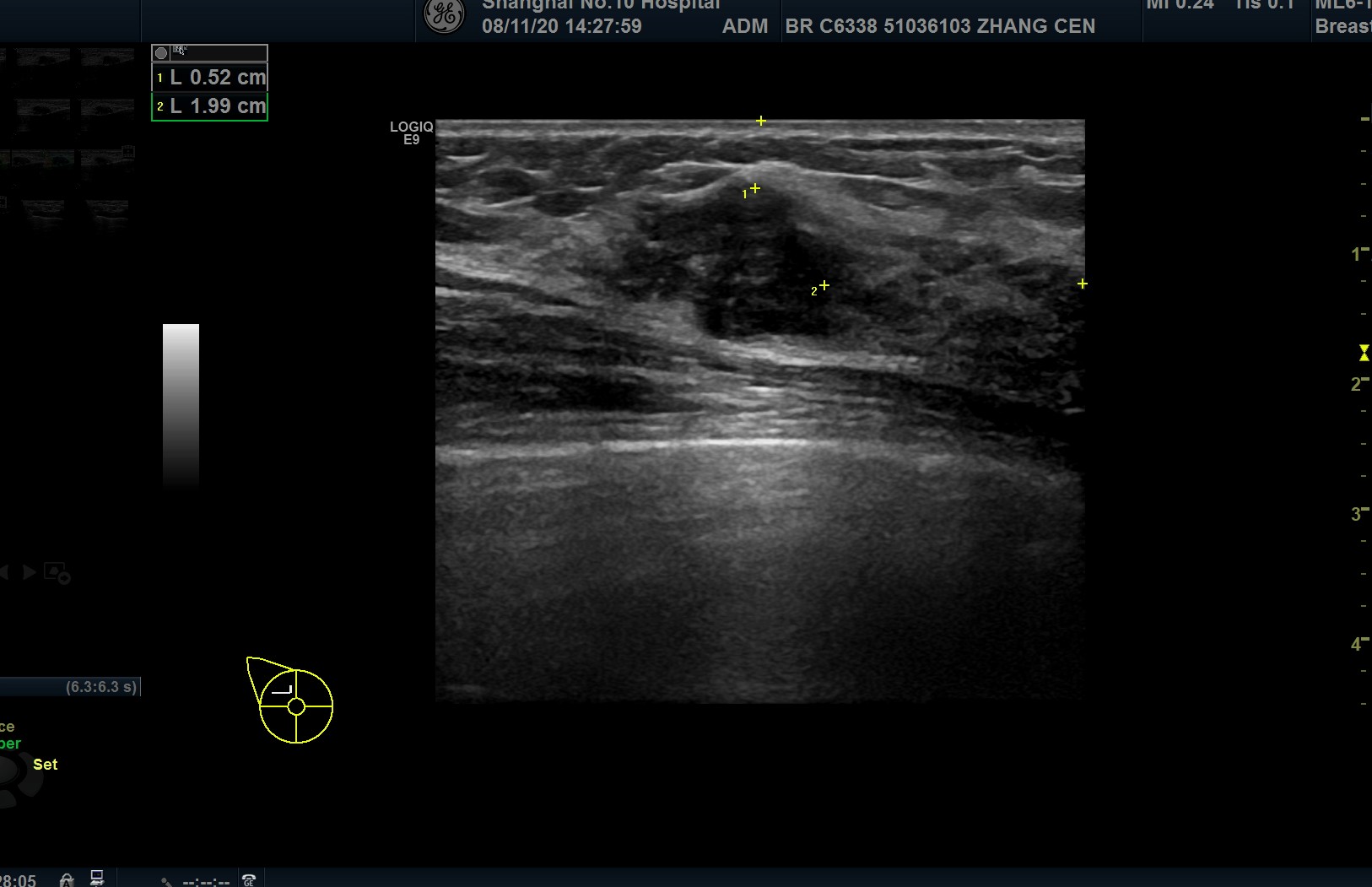

Supplement: Supplementary file 1 [file DataSheet_1.zip › 3/700168_杨柳依/杨柳依_44703258.jpg]

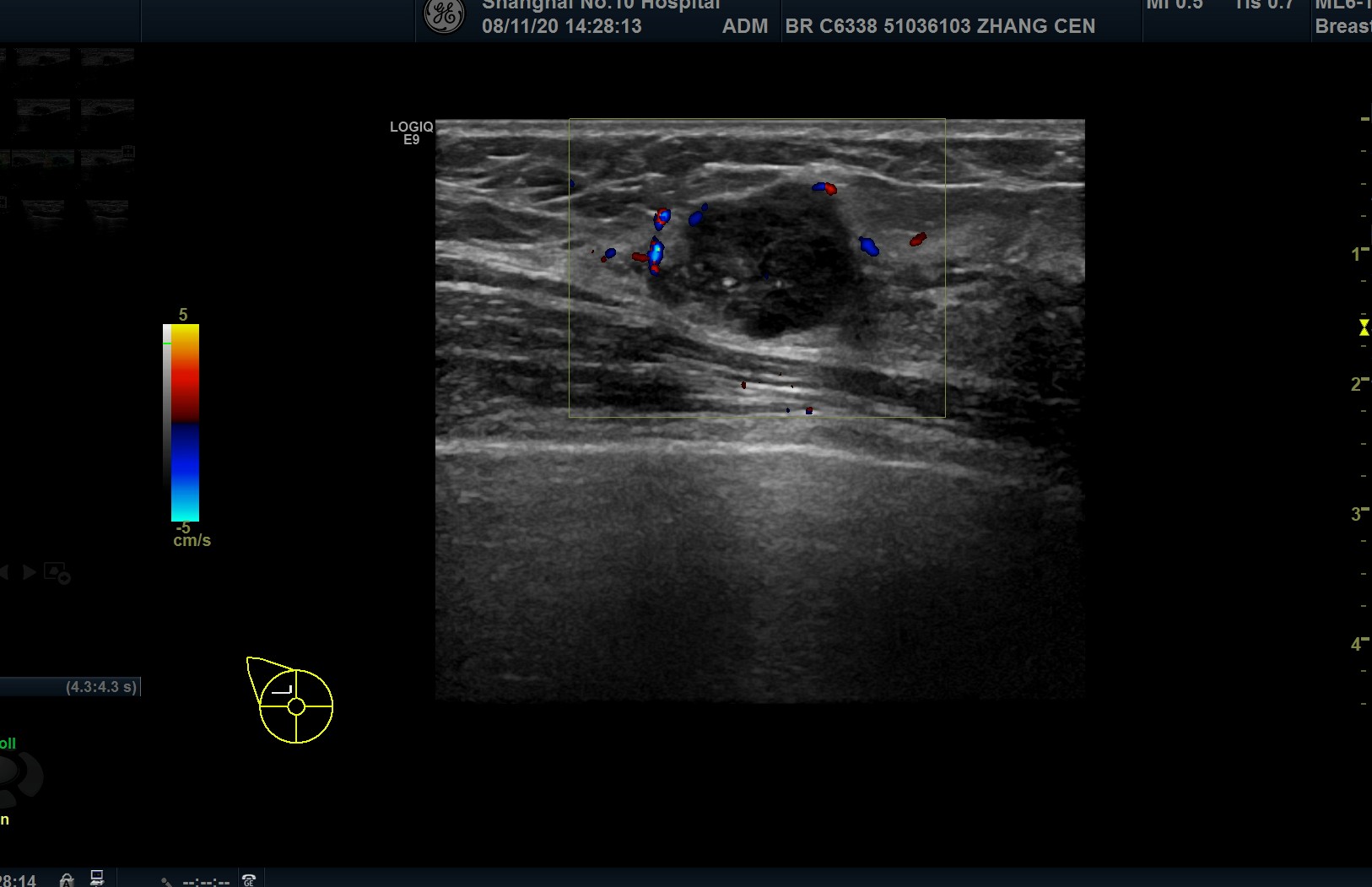

Supplement: Supplementary file 1 [file DataSheet_1.zip › 3/700168_杨柳依/杨柳依_44703261.jpg]

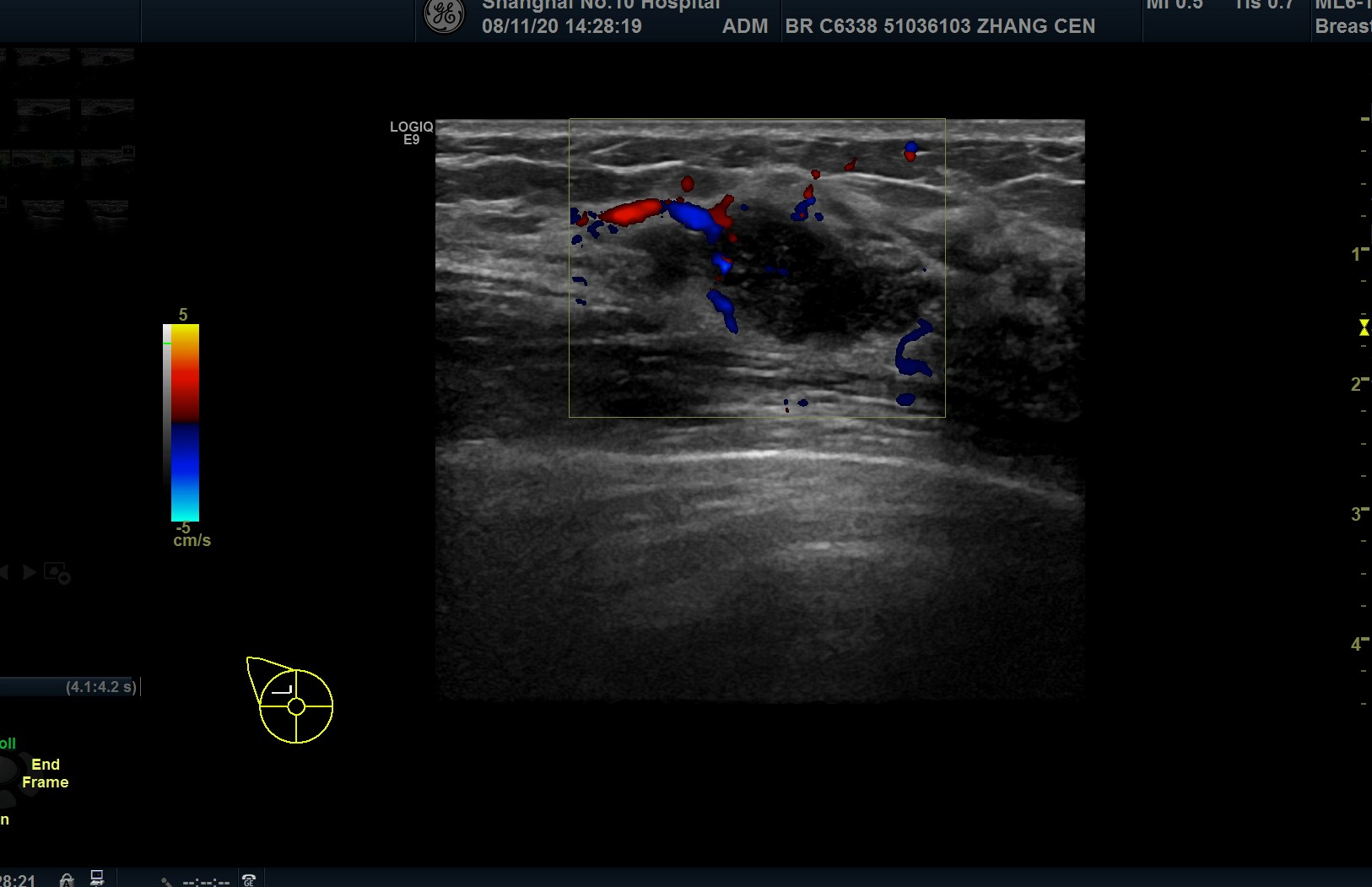

Supplement: Supplementary file 1 [file DataSheet_1.zip › 3/700168_杨柳依/杨柳依_44703269.jpg]

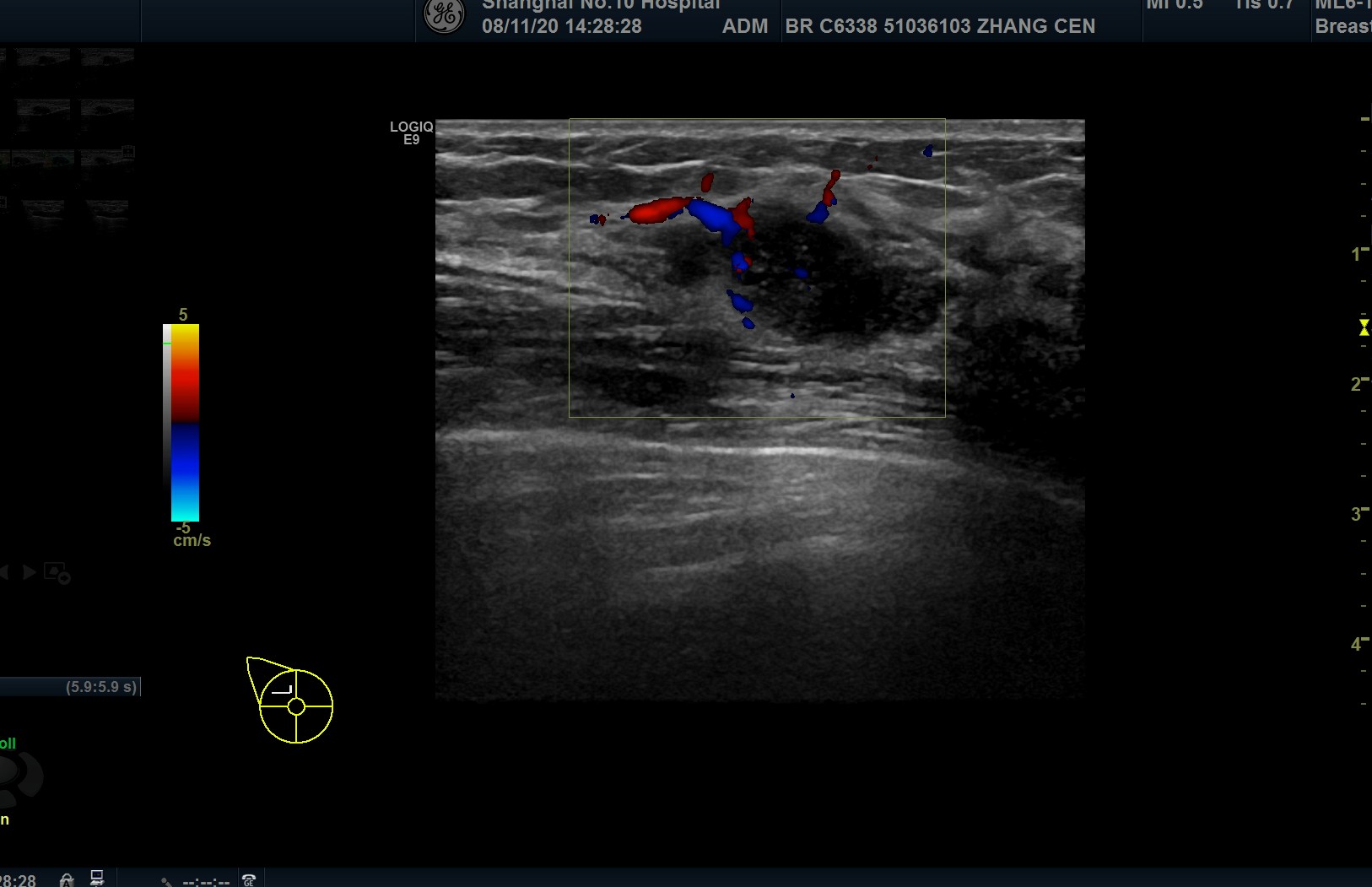

Supplement: Supplementary file 1 [file DataSheet_1.zip › 3/700168_杨柳依/杨柳依_44703274.jpg]

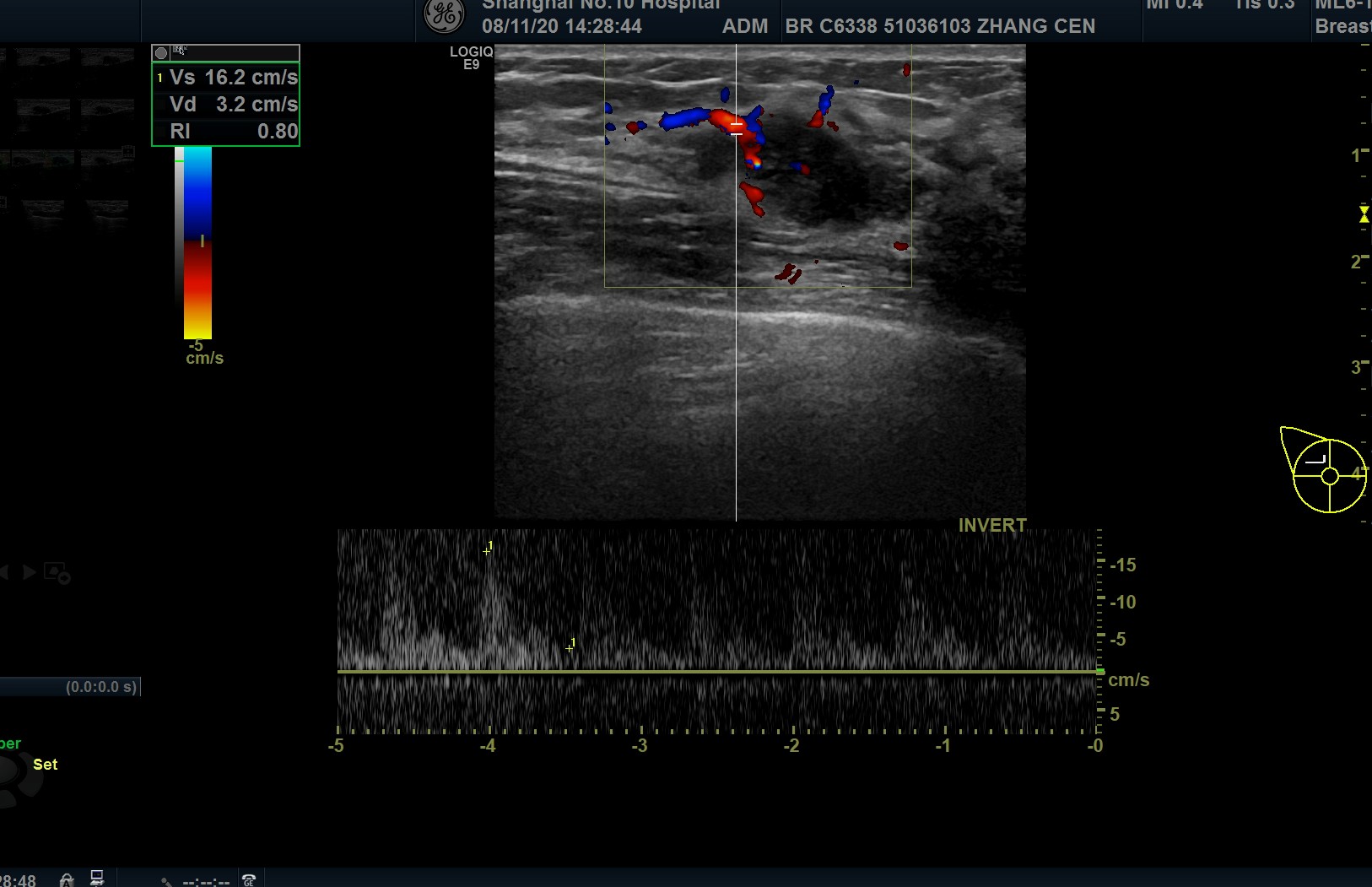

Supplement: Supplementary file 1 [file DataSheet_1.zip › 3/700168_杨柳依/杨柳依_44703284.jpg]

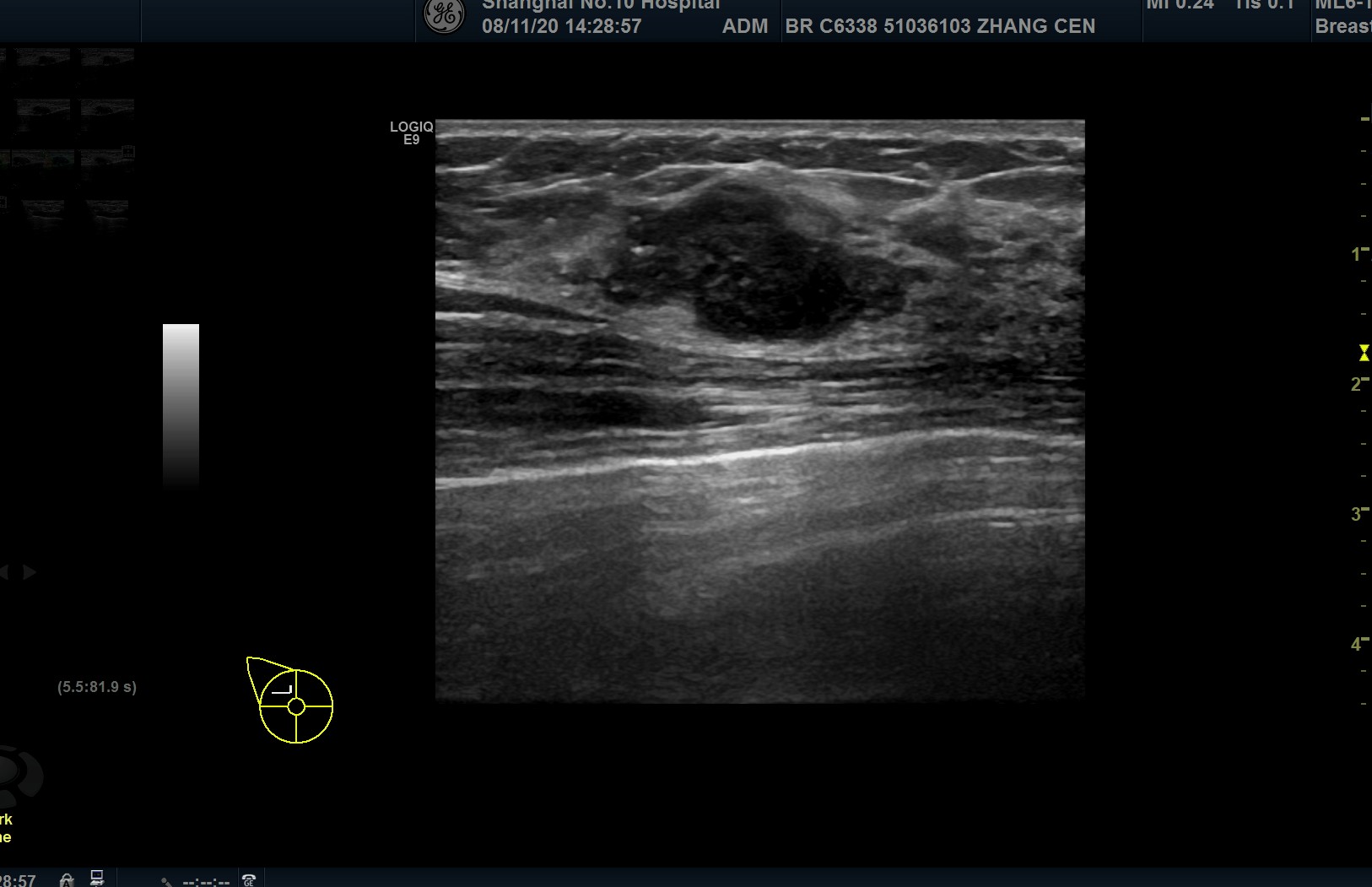

Supplement: Supplementary file 1 [file DataSheet_1.zip › 3/700168_杨柳依/杨柳依_44703286.jpg]

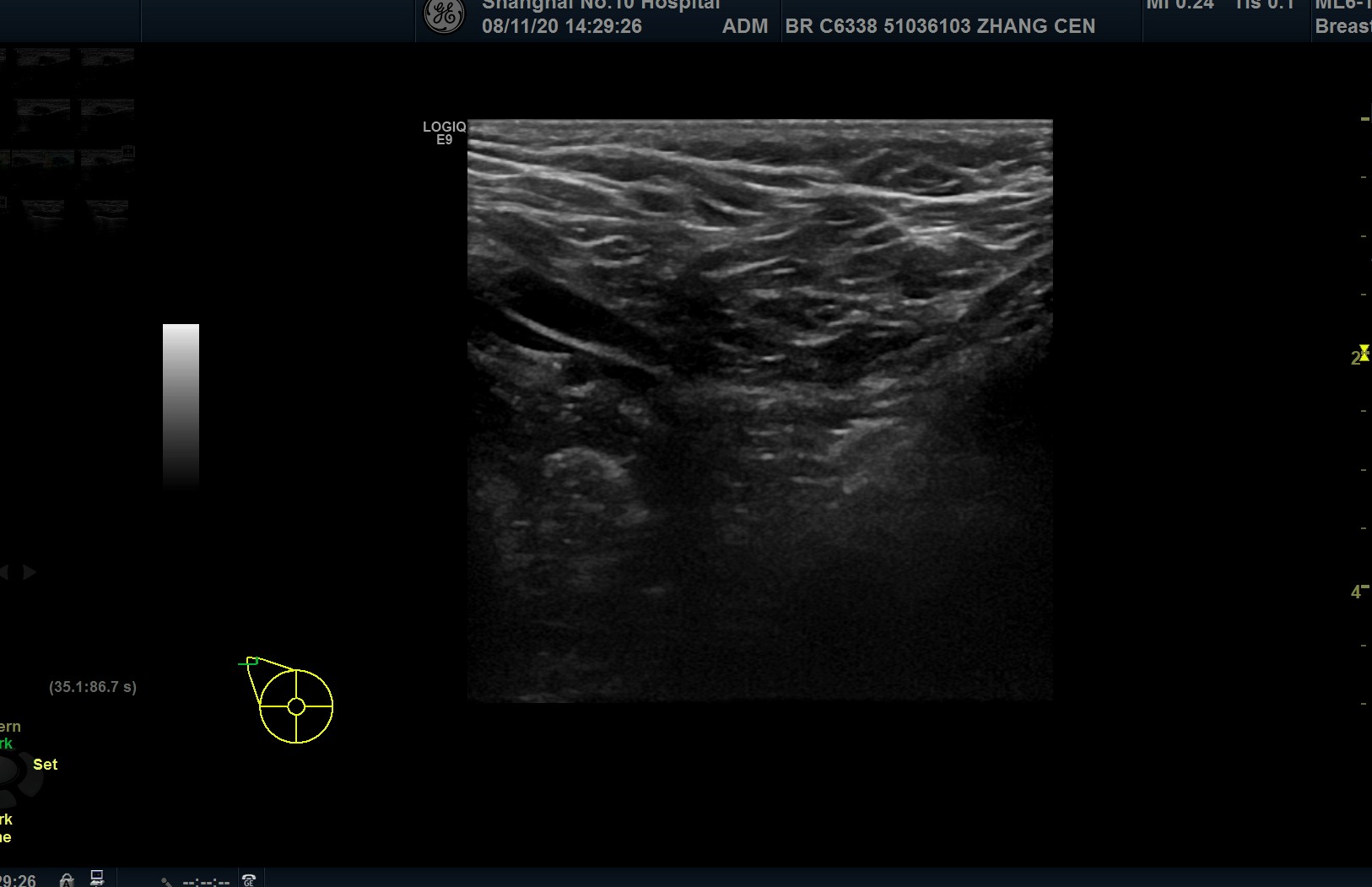

Supplement: Supplementary file 1 [file DataSheet_1.zip › 3/700168_杨柳依/杨柳依_44703302.jpg]

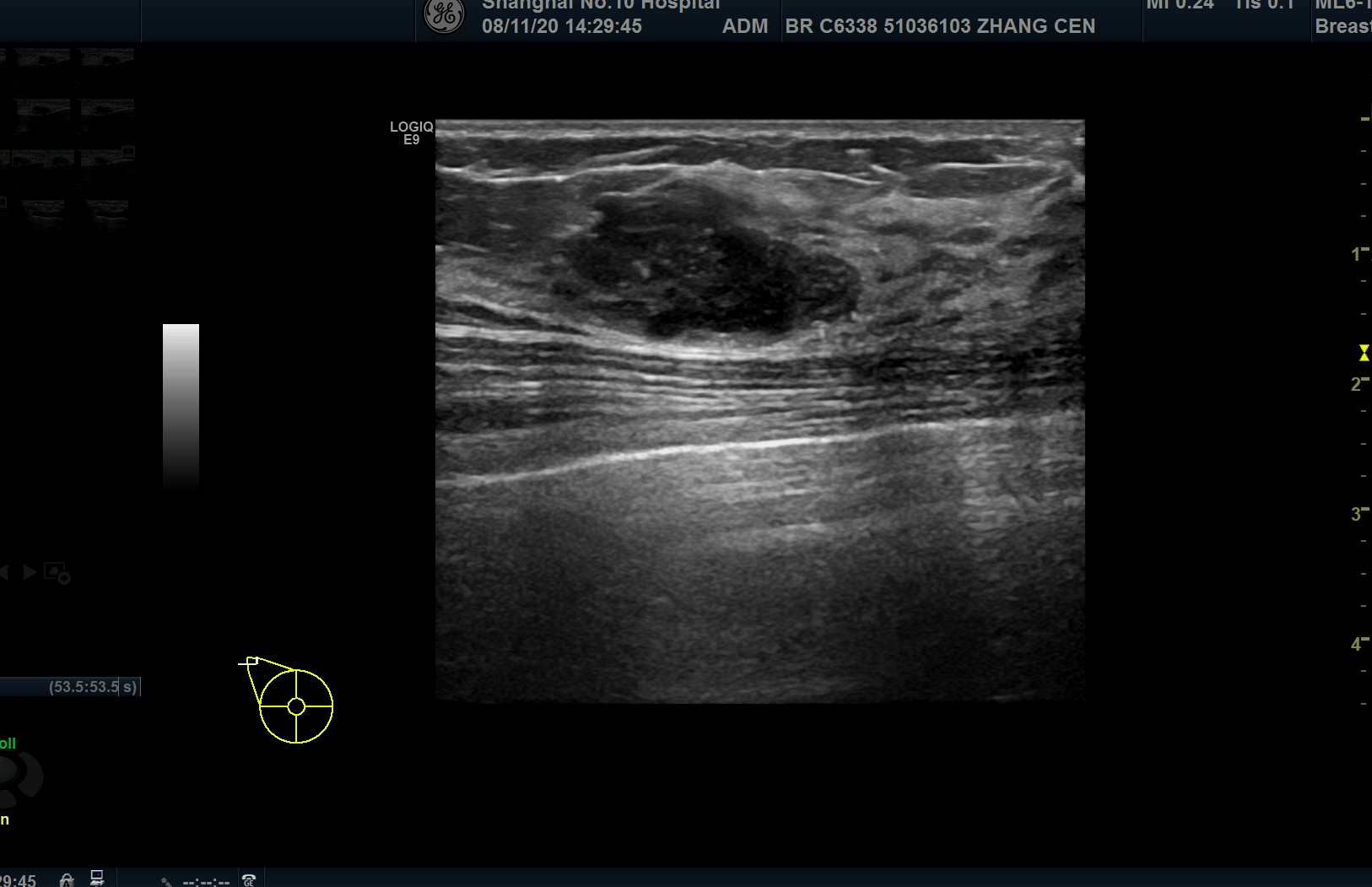

Supplement: Supplementary file 1 [file DataSheet_1.zip › 3/700168_杨柳依/杨柳依_44703315.jpg]

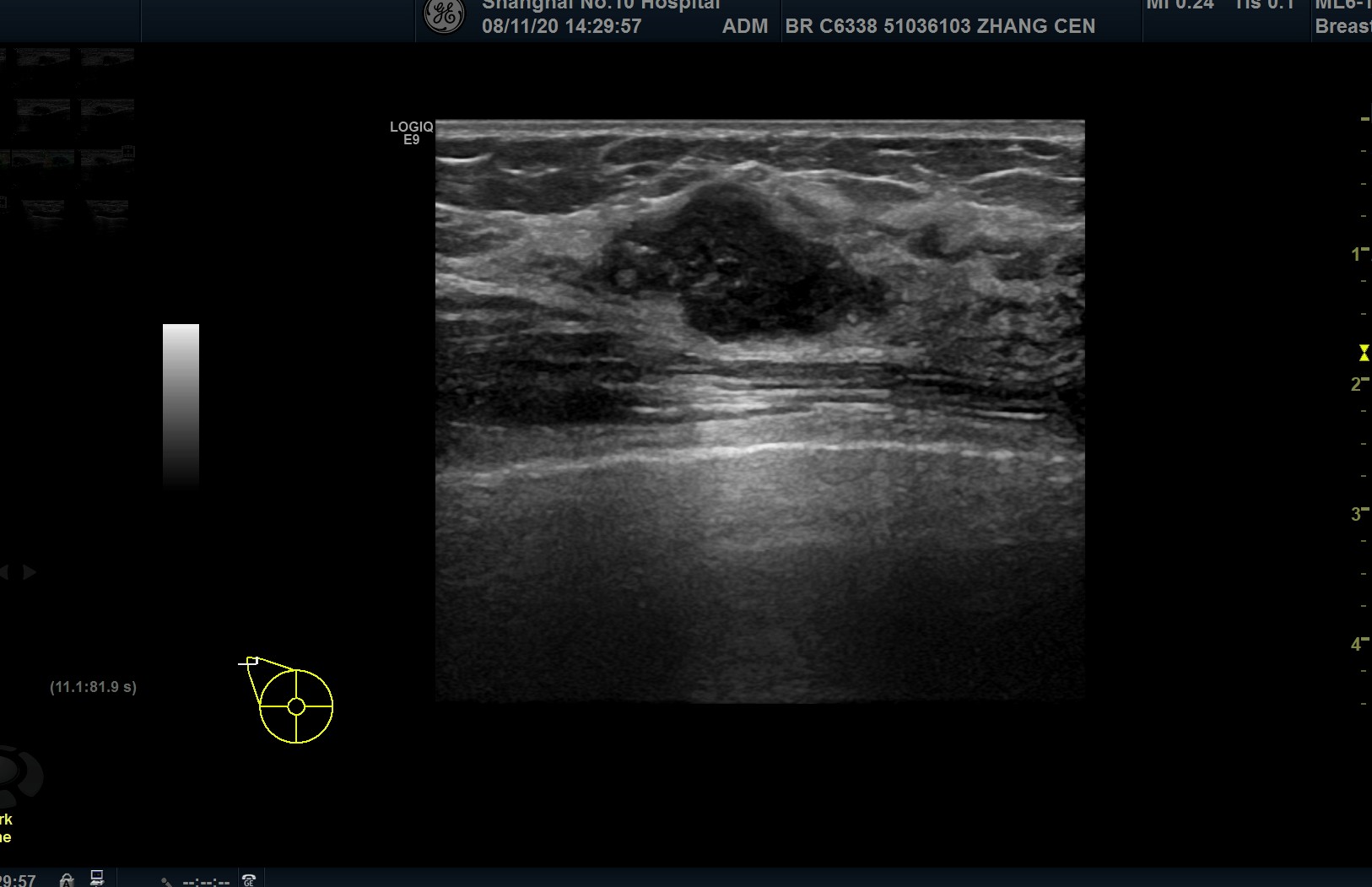

Supplement: Supplementary file 1 [file DataSheet_1.zip › 3/700168_杨柳依/杨柳依_44703320.jpg]

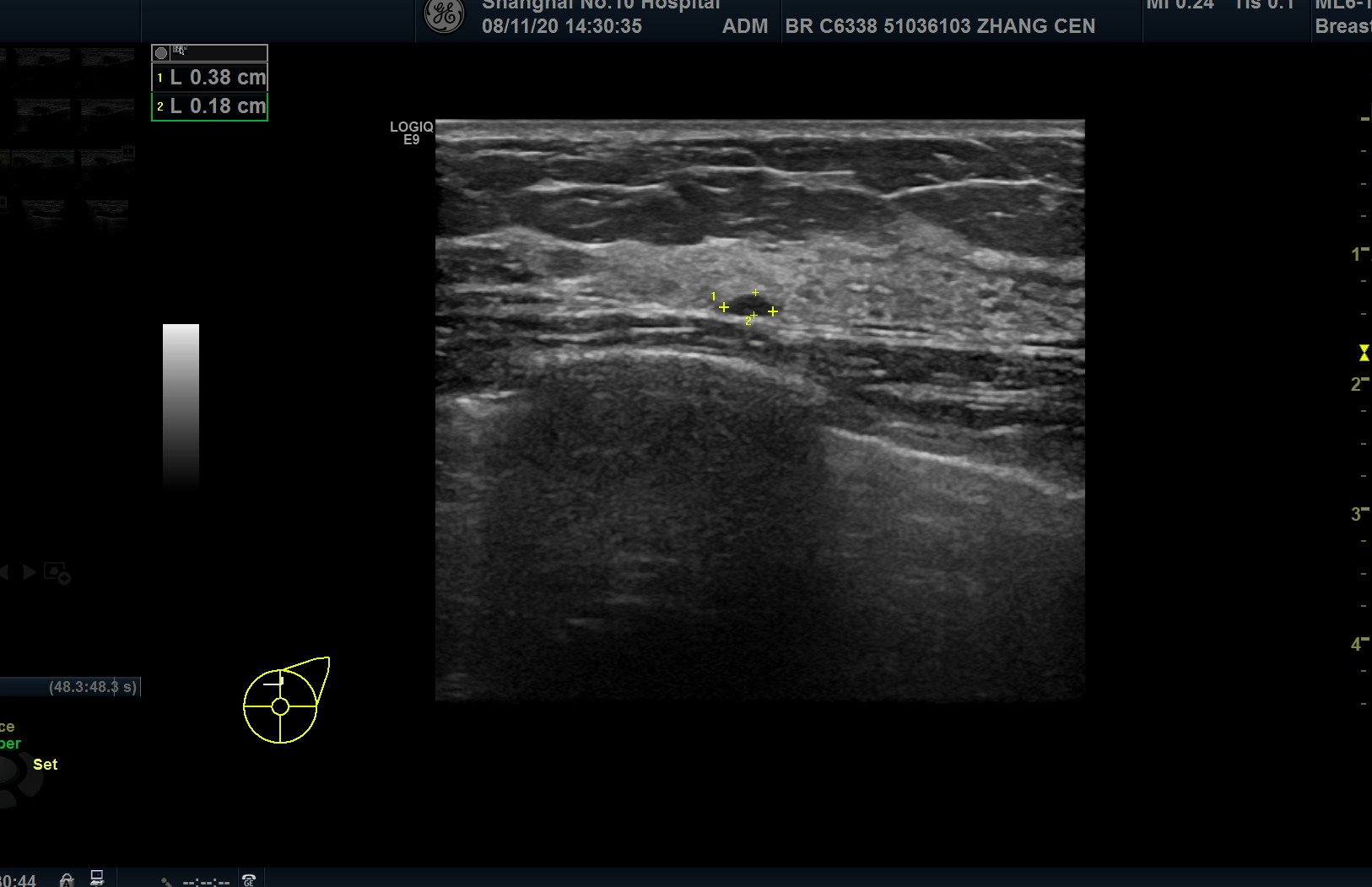

Supplement: Supplementary file 1 [file DataSheet_1.zip › 3/700168_杨柳依/杨柳依_44703337.jpg]

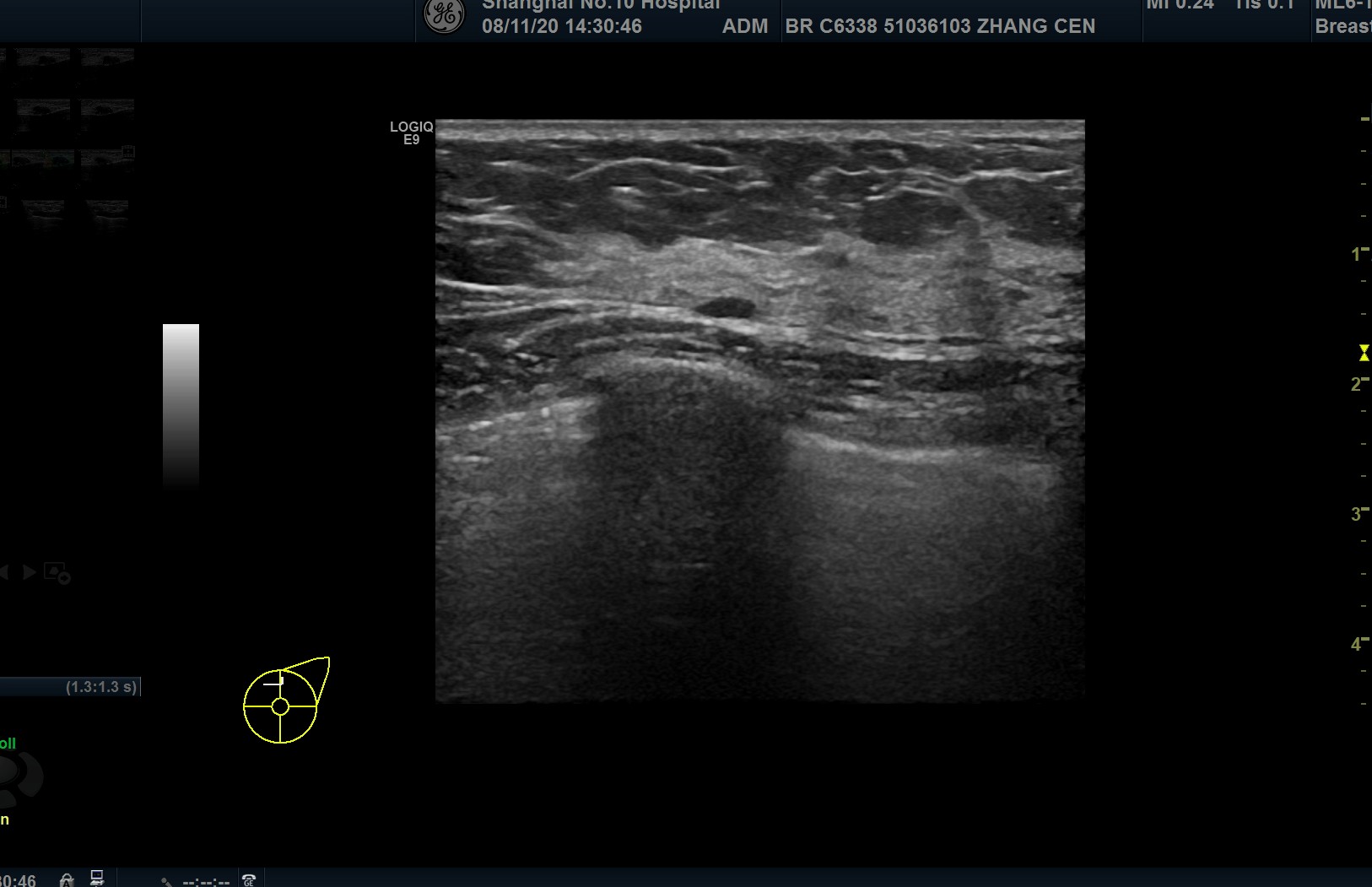

Supplement: Supplementary file 1 [file DataSheet_1.zip › 3/700168_杨柳依/杨柳依_44703341.jpg]

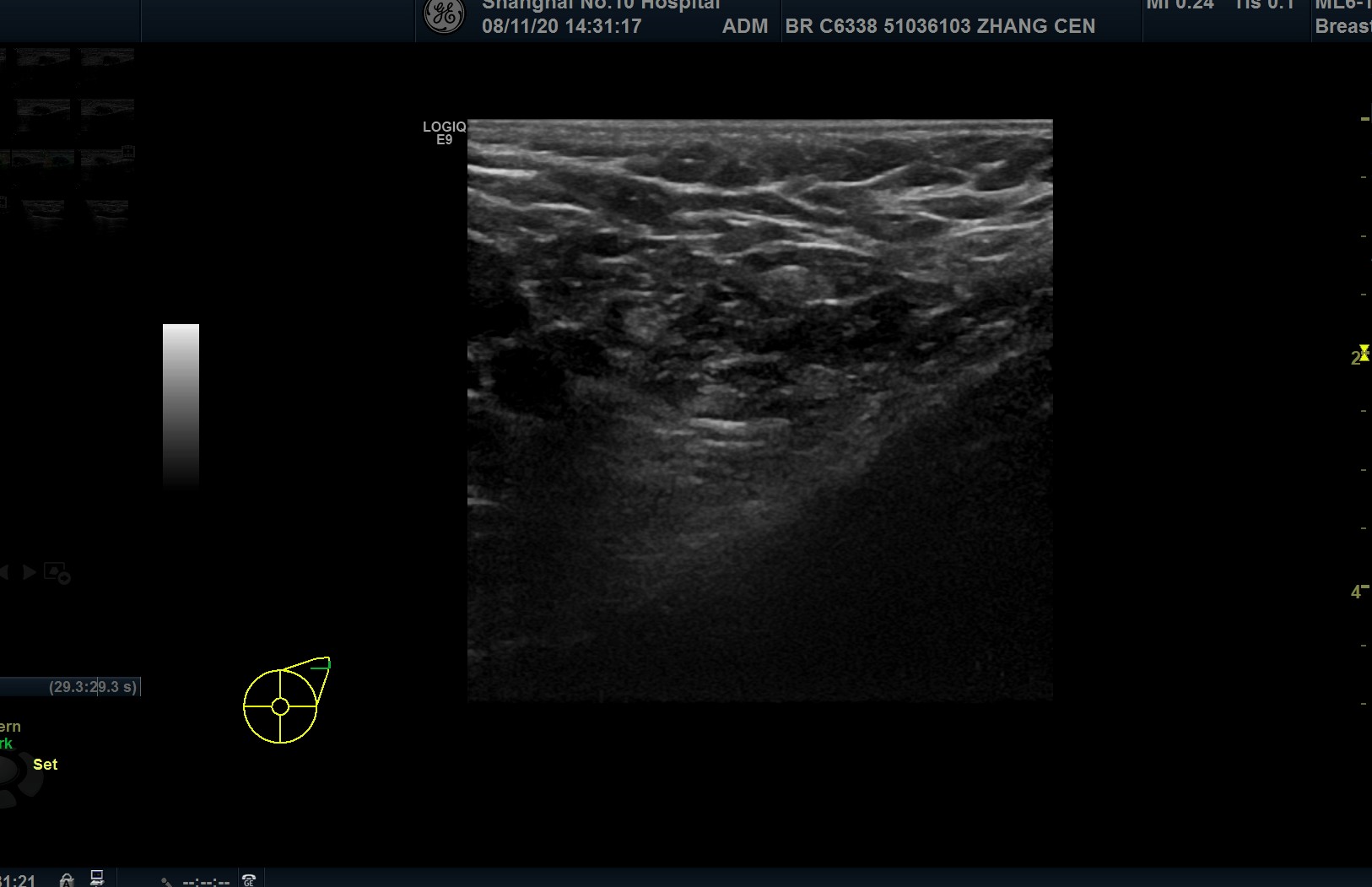

Supplement: Supplementary file 1 [file DataSheet_1.zip › 3/700168_杨柳依/杨柳依_44703355.jpg]

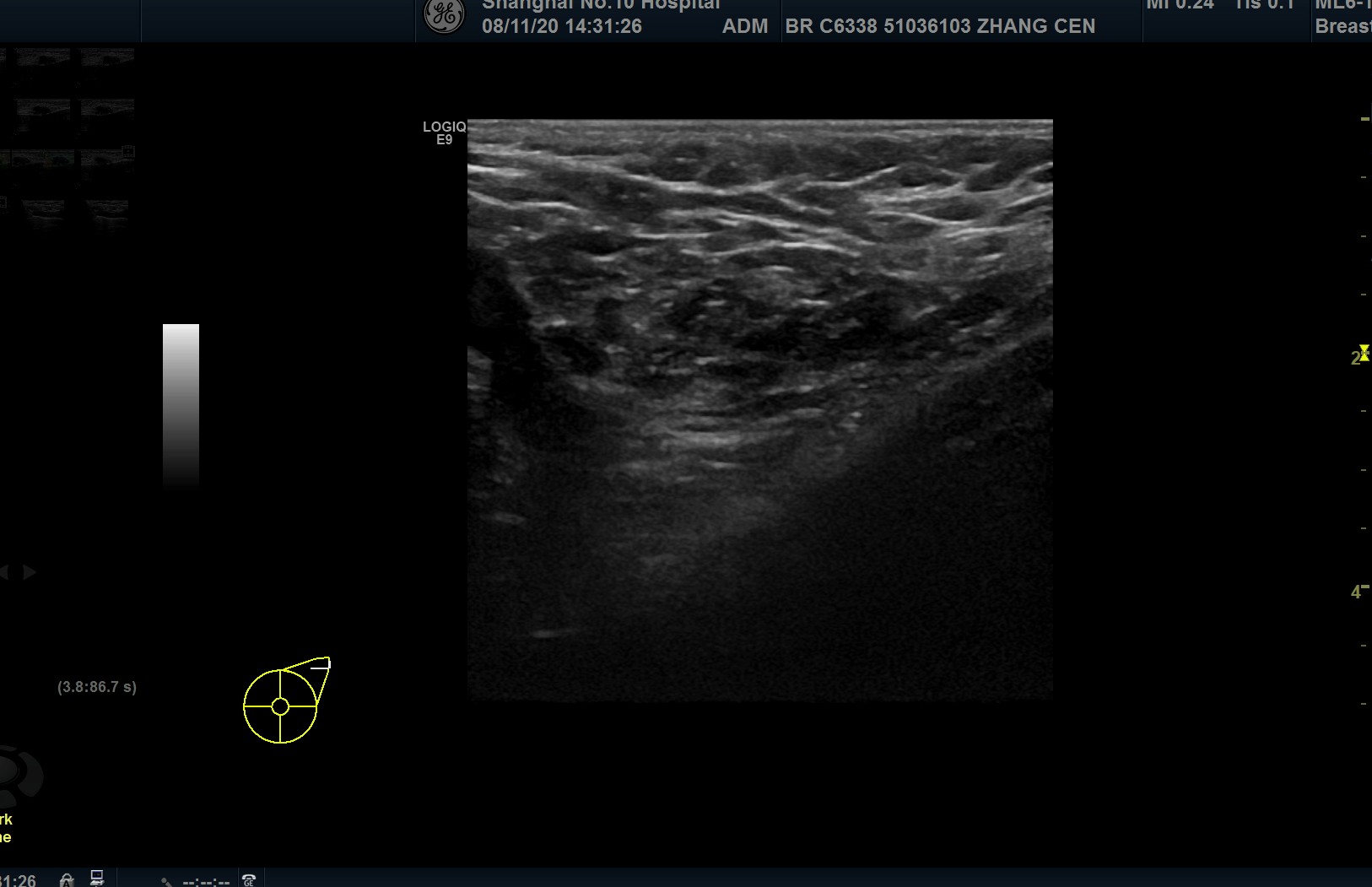

Supplement: Supplementary file 1 [file DataSheet_1.zip › 3/700168_杨柳依/杨柳依_44703357.jpg]

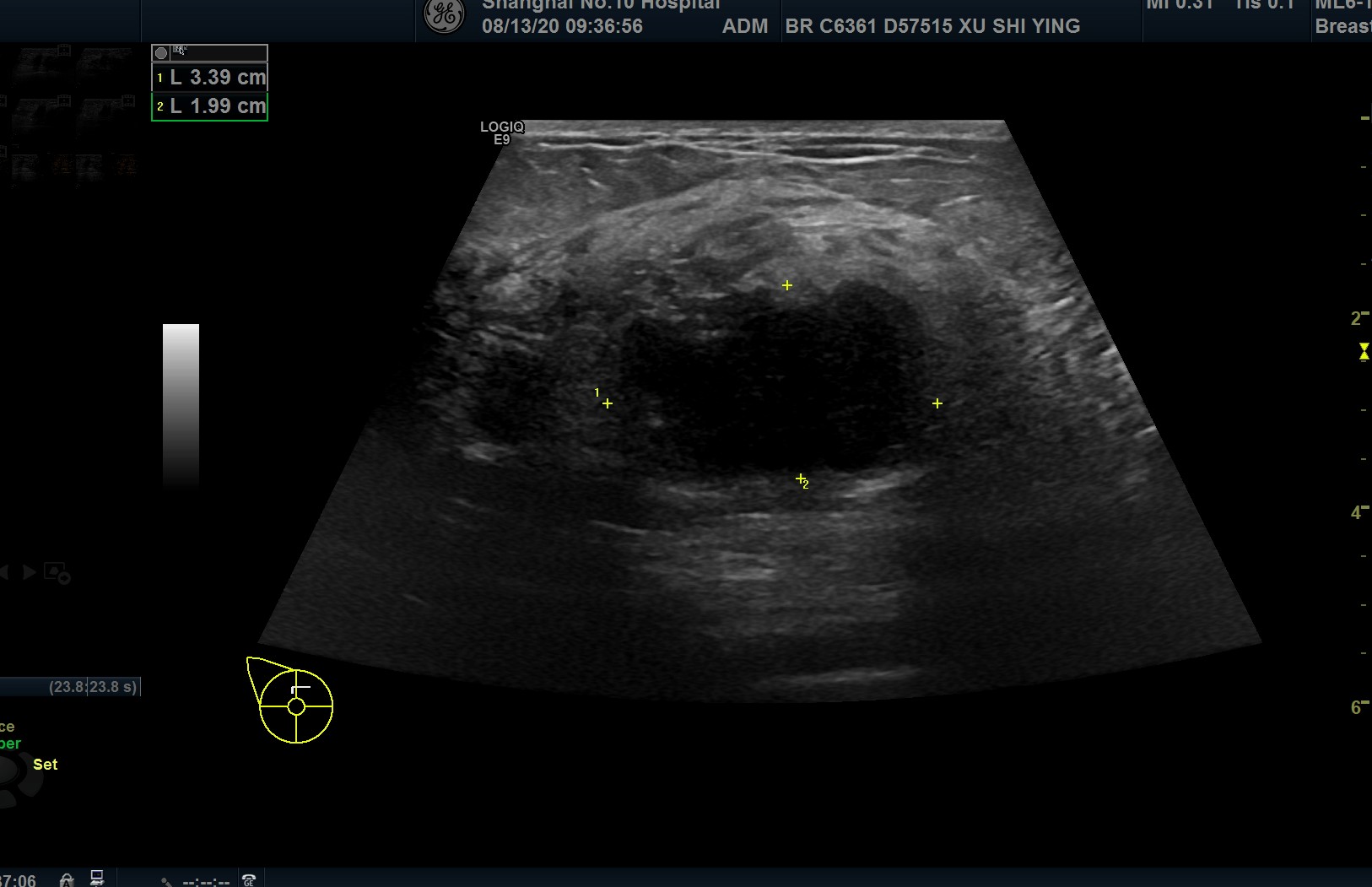

Supplement: Supplementary file 1 [file DataSheet_1.zip › 3/701141_沈建丽/沈建丽_44724679.jpg]

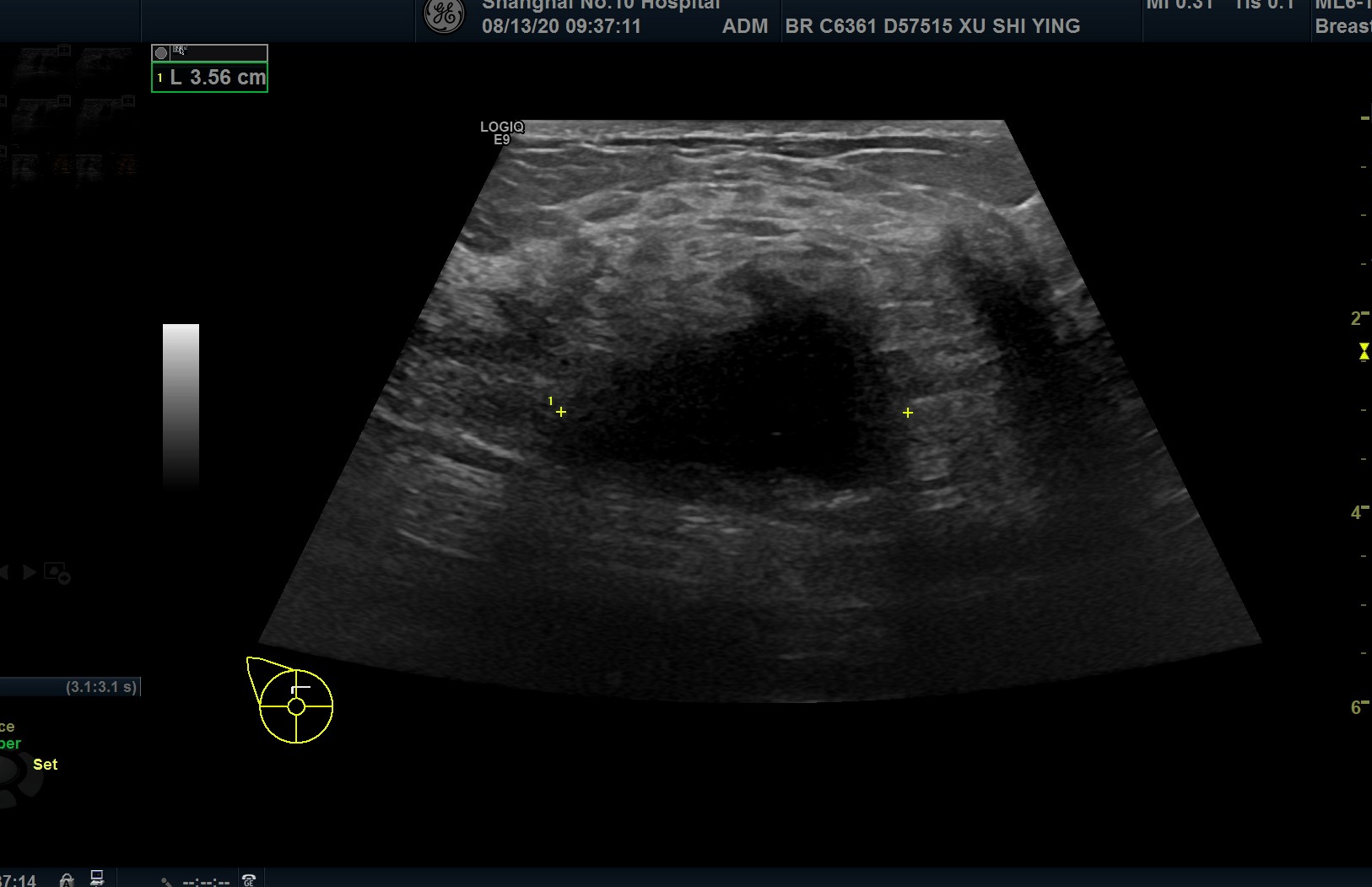

Supplement: Supplementary file 1 [file DataSheet_1.zip › 3/701141_沈建丽/沈建丽_44724688.jpg]

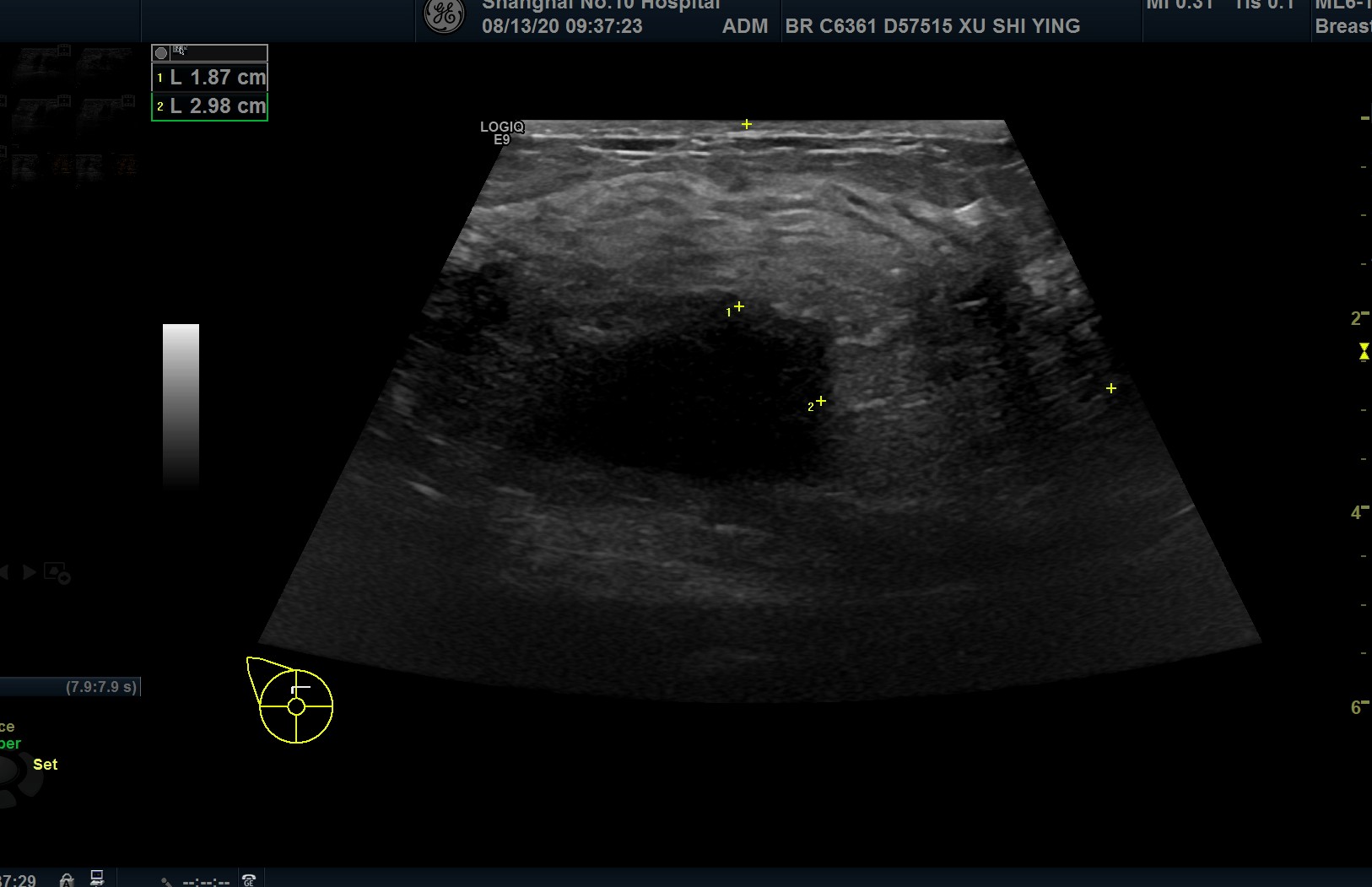

Supplement: Supplementary file 1 [file DataSheet_1.zip › 3/701141_沈建丽/沈建丽_44724701.jpg]

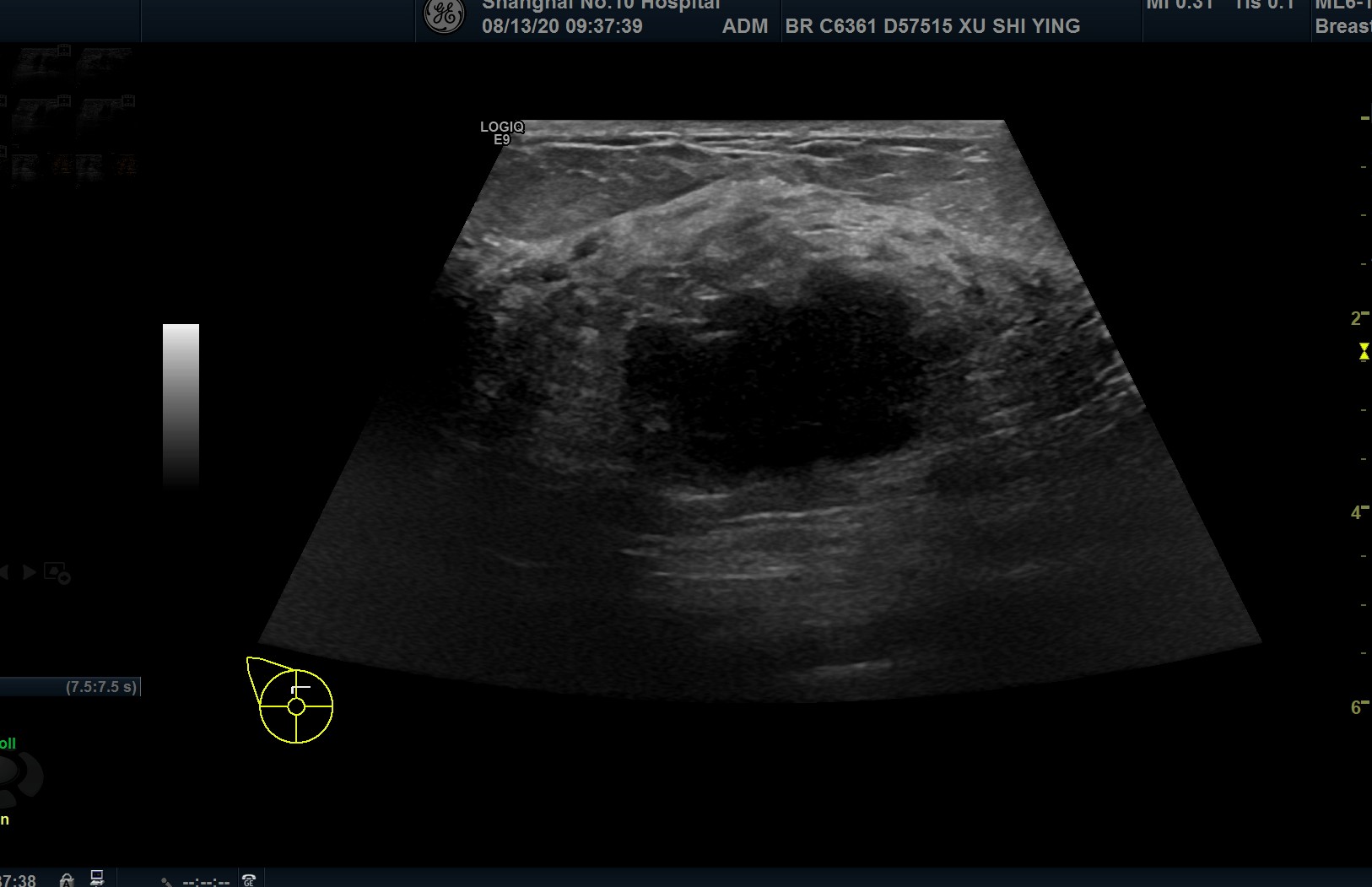

Supplement: Supplementary file 1 [file DataSheet_1.zip › 3/701141_沈建丽/沈建丽_44724710.jpg]

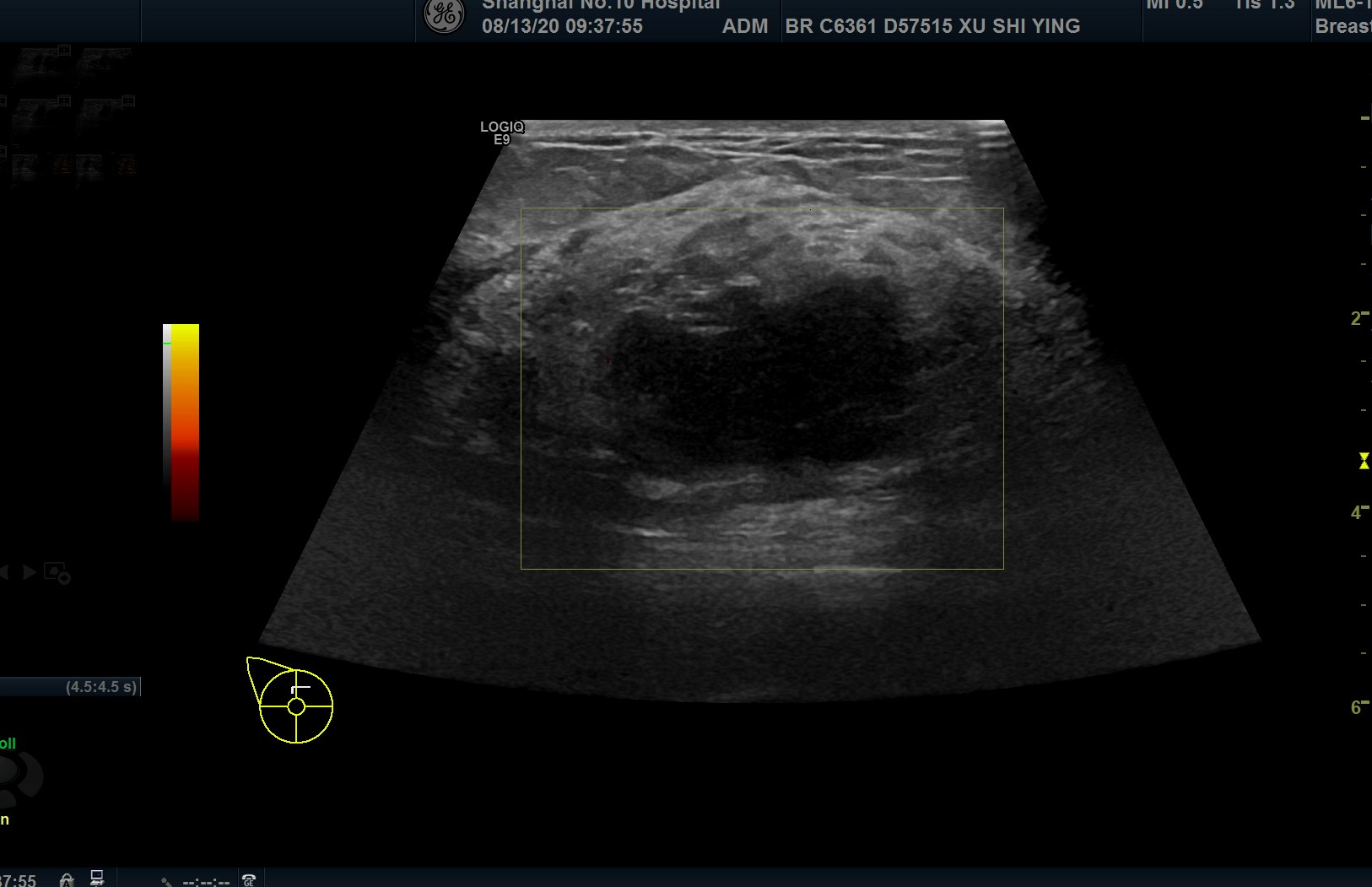

Supplement: Supplementary file 1 [file DataSheet_1.zip › 3/701141_沈建丽/沈建丽_44724730.jpg]

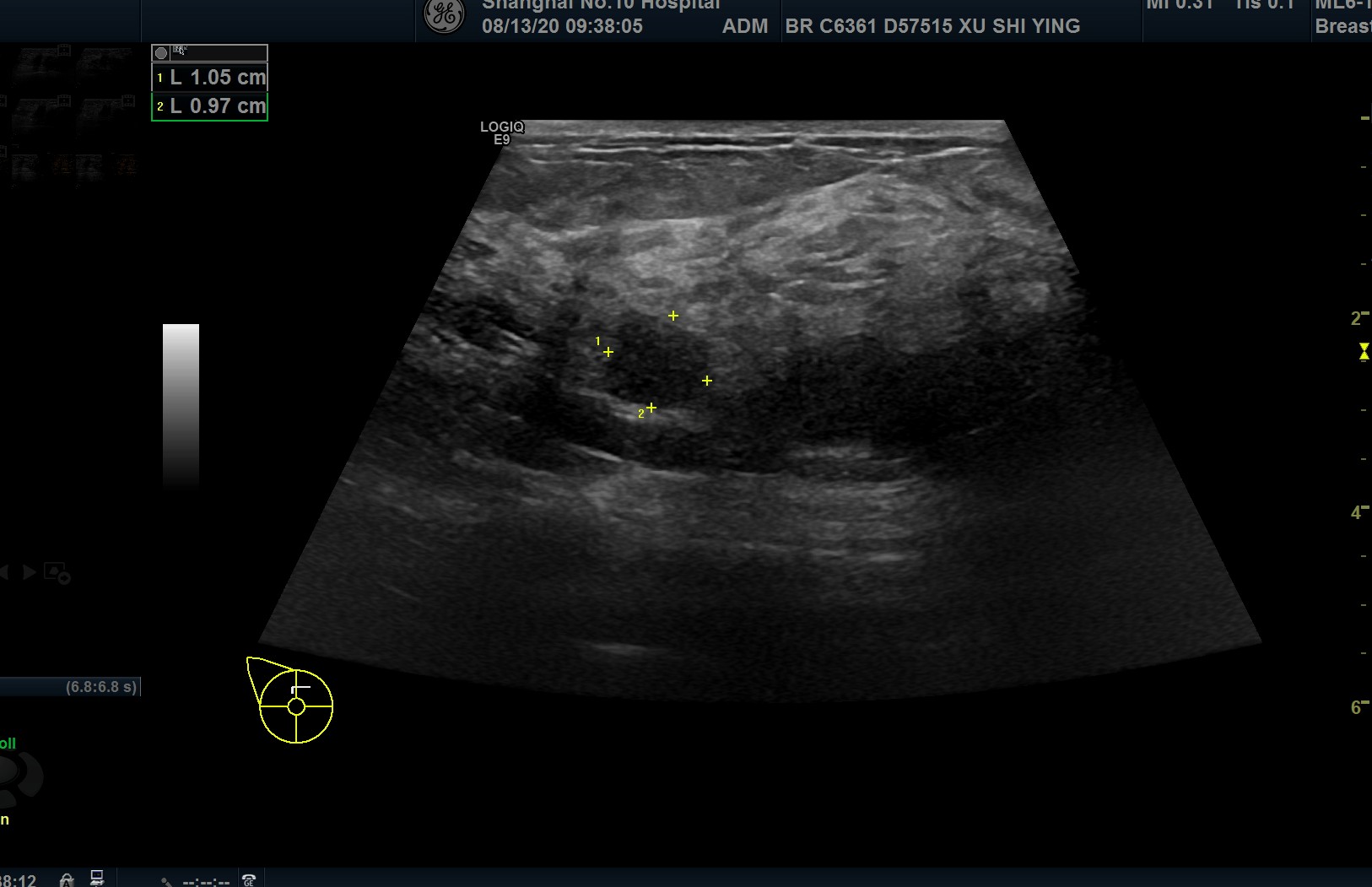

Supplement: Supplementary file 1 [file DataSheet_1.zip › 3/701141_沈建丽/沈建丽_44724745.jpg]

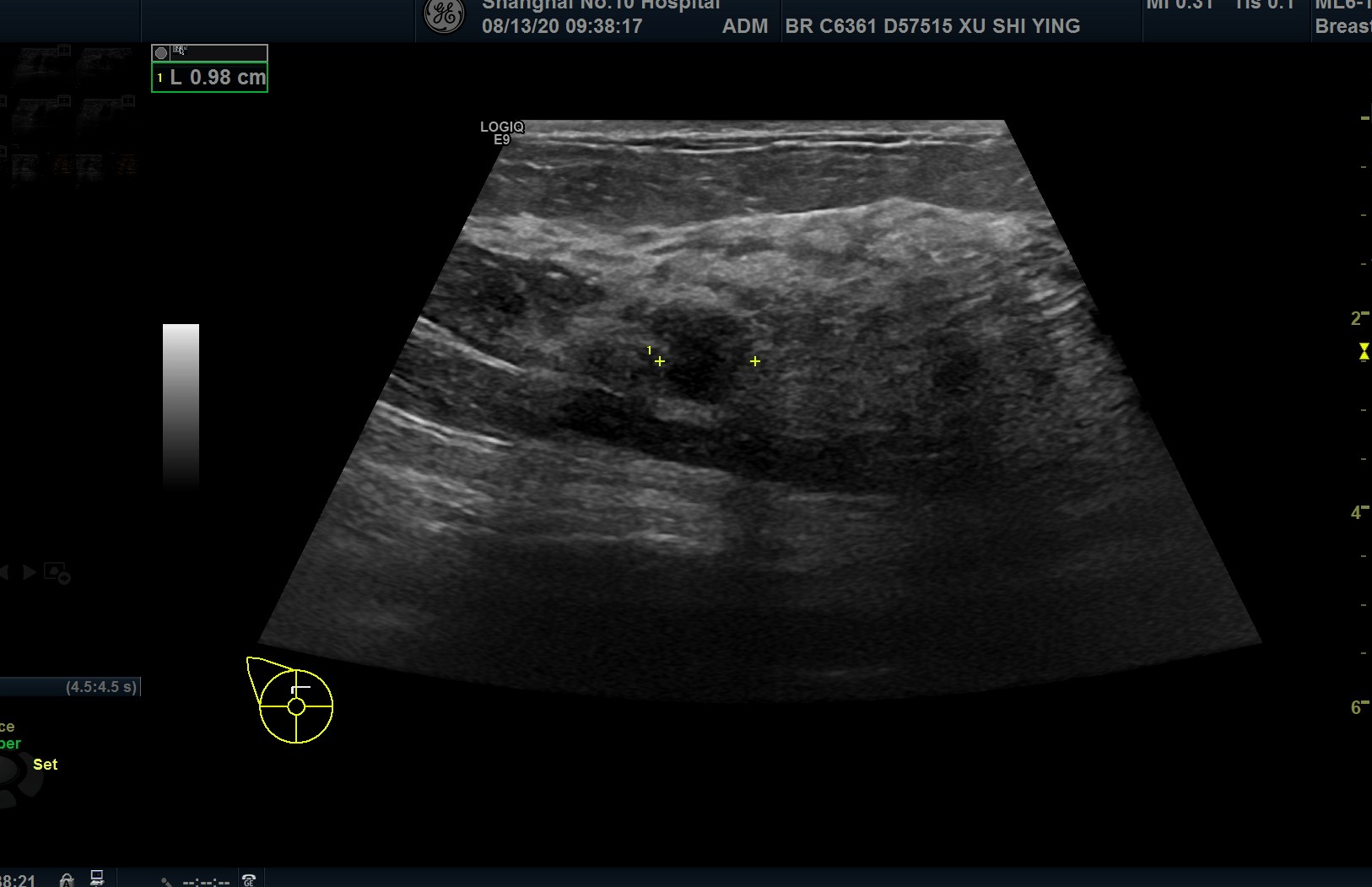

Supplement: Supplementary file 1 [file DataSheet_1.zip › 3/701141_沈建丽/沈建丽_44724756.jpg]

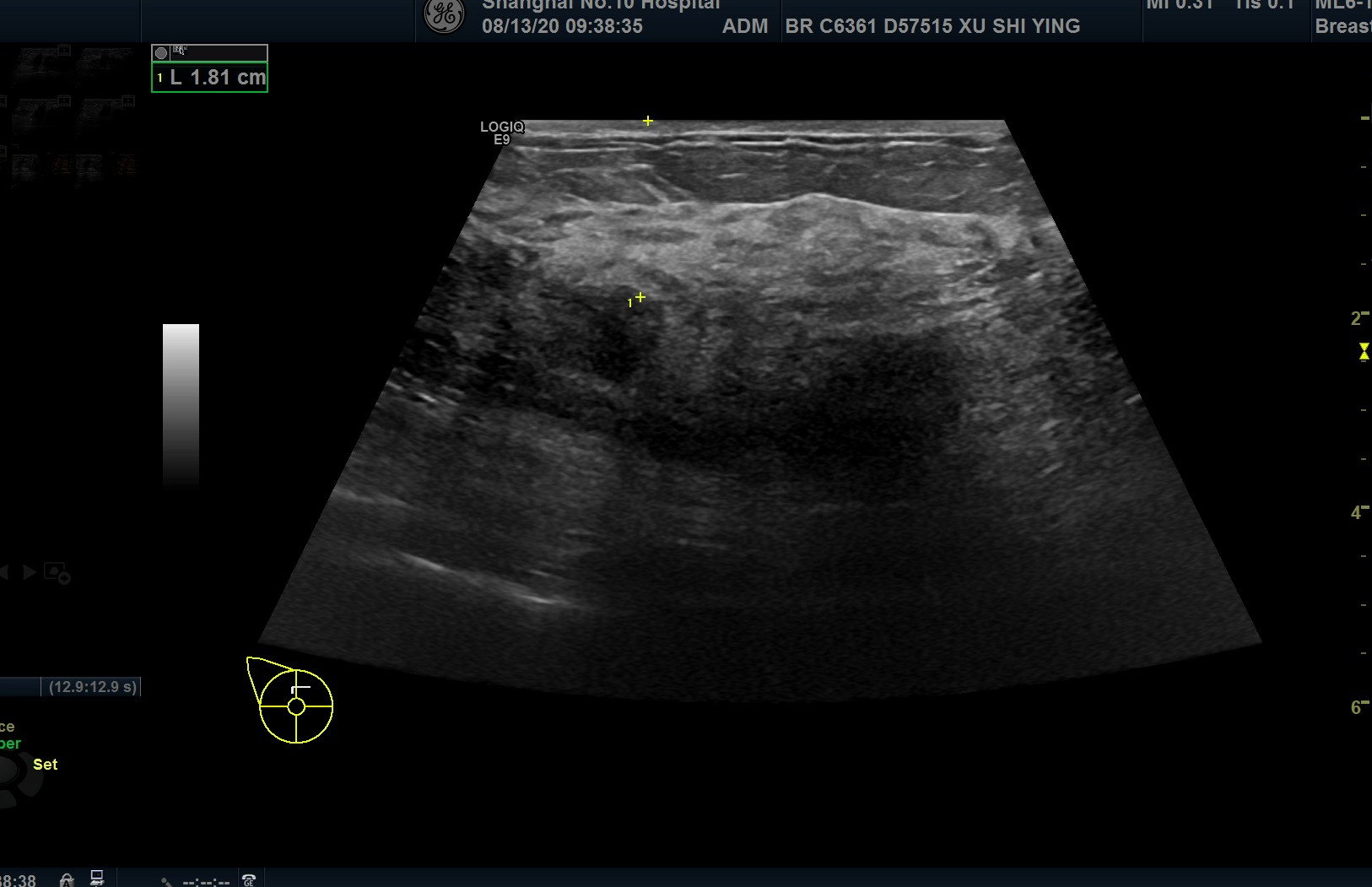

Supplement: Supplementary file 1 [file DataSheet_1.zip › 3/701141_沈建丽/沈建丽_44724769.jpg]

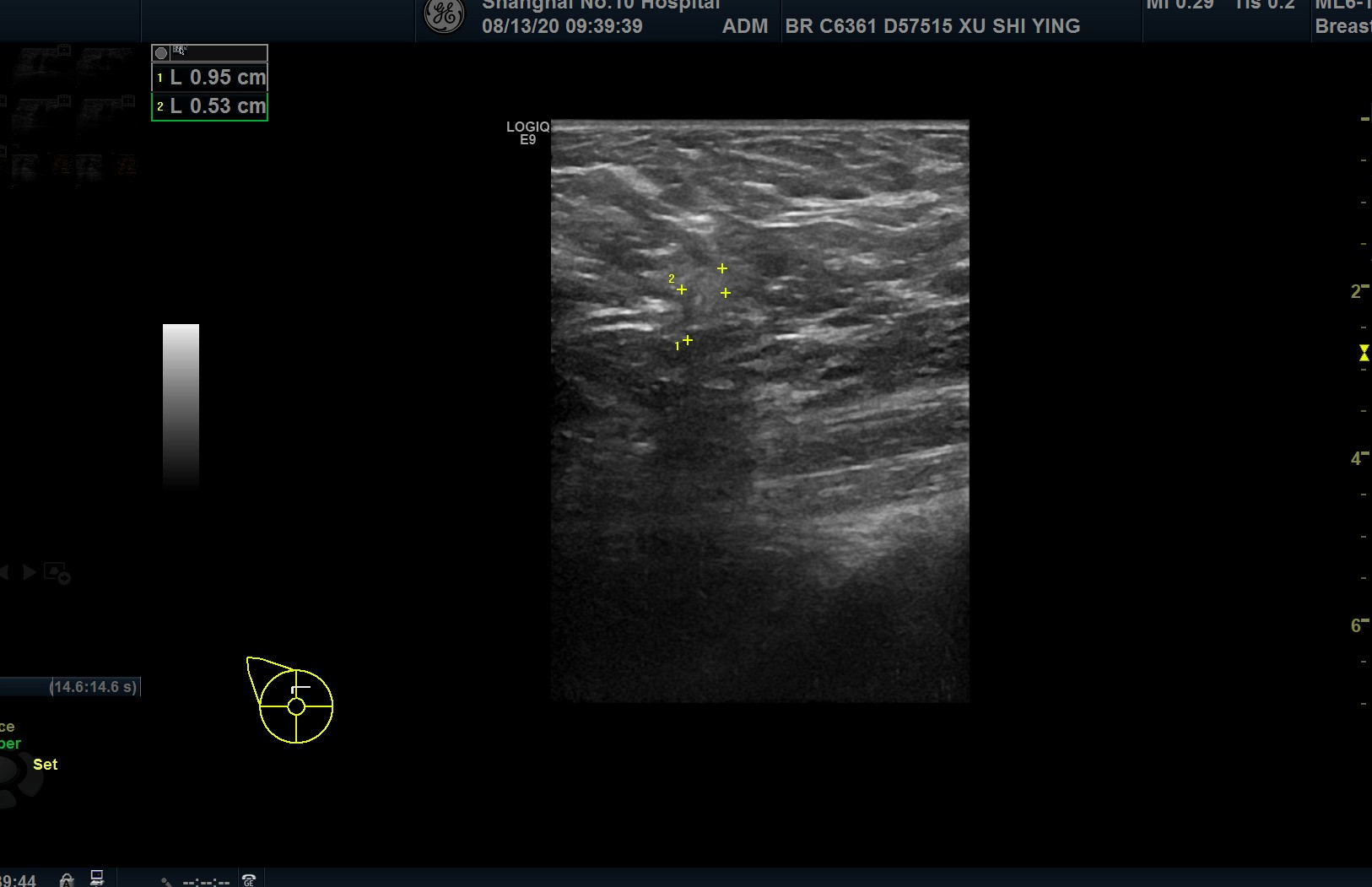

Supplement: Supplementary file 1 [file DataSheet_1.zip › 3/701141_沈建丽/沈建丽_44724811.jpg]

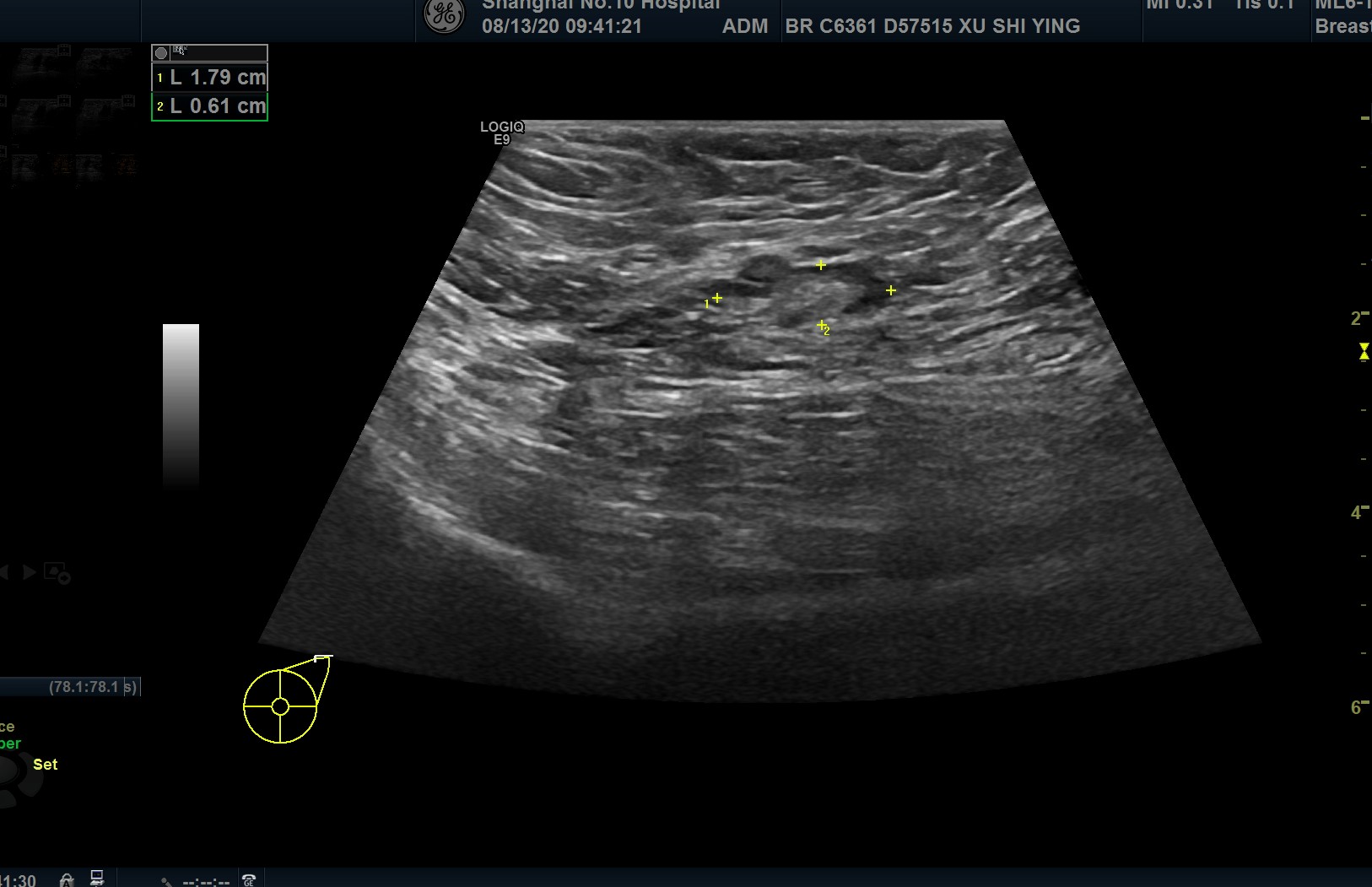

Supplement: Supplementary file 1 [file DataSheet_1.zip › 3/701141_沈建丽/沈建丽_44724872.jpg]

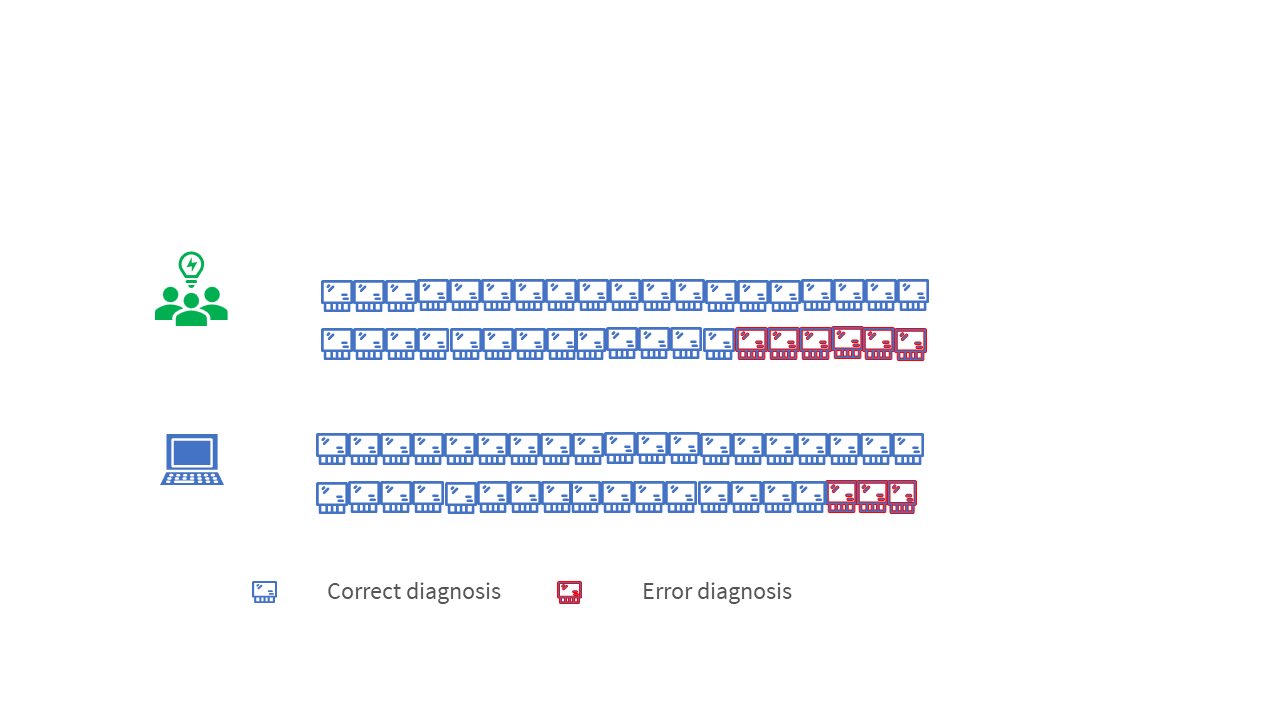

Supplement: Supplementary file 2 [file Image_1.tif]
